# Synthesis and Biological Evaluation of Novel Selenyl and Sulfur-l-Dopa Derivatives as Potential Anti-Parkinson’s Disease Agents

**DOI:** 10.3390/biom9060239

**Published:** 2019-06-18

**Authors:** Antonio Di Stefano, Lisa Marinelli, Piera Eusepi, Michele Ciulla, Stefania Fulle, Ester Sara Di Filippo, Laura Magliulo, Giuseppe Di Biase, Ivana Cacciatore

**Affiliations:** 1 Department of Pharmacy, University ”G. d’Annunzio” Chieti-Pescara, 66100 Chieti, Italy; antonio.distefano@unich.it (A.D.S); l.marinelli@unich.it (L.M.); piera.eusepi@unich.it (P.E.); michele.ciulla@unich.it (M.C.); giuseppe.dibiase@unich.it (G.D.B.); 2 Department of Neuroscience, Imaging and Clinical Sciences, University ”G. d’Annunzio” Chieti-Pescara, 66100 Chieti, Italy; stefania.fulle@unich.it (S.F.); es.difilippo@unich.it (E.S.D.F.); laura.magliulo@unich.it (L.M.)

**Keywords:** l-dopa, Parkinson’s disease, selenium compounds, sulfur-derivatives

## Abstract

Parkinson’s disease (PD) is a neurodegenerative disorder characterized by loss of dopaminergic neurons at level of *substantia nigra pars compacta*. To date, there is no cure for this pathology, except for some drugs able to alleviate the symptoms of PD. In this paper we report the synthesis and biological evaluation of novel sulfur- and selenyl-l-Dopa (LD) derivatives (**SP1–6**) obtained through the amide junction between the amino group of LD and carboxylic moiety of sulfur- and selenyl-organic compounds, which are commercially available. Biological activity was evaluated on human undifferentiated and retinoic acid/phorbol myristyl acetate (RA/PMA)-differentiated SY-SH5Y neuroblastoma cell line using 3-(4,5-dimethylthiazol-2-yl)-2,5-diphenyltetrazolium bromide (MTT) assay. Antioxidant activity against oxidative stress was measured using nitroblue tetrazolium (NBT) and 2’,7’-dichlorodihydrofluorescein diacetate (H_2_DCFDA) assays. Finally, physico-chemical characterization and plasma stability studies of **SP1–6** were also performed. Biological data revealed that **SP6** has a significant protective action against the neurotoxic action of 6-hydroxydopamine (6-OHDA) and H_2_O_2_ in a RA/PMA-differentiated SY-SH5Y neuroblastoma cell line that proved to be an effective antioxidant and protective compound. **SP6**, endowed with a lipophilic nature, low molecular weight, and plasma stability, can easily cross biological membranes via passive diffusion such as through the blood–brain barrier. **SP6** has great potential for developing novel pharmacological approach for neurodegenerative diseases, such as PD. Further studies will help define its exact antioxidant mechanism and determine whether the neuroprotective action is mediated or modulated by glutathione peroxidase (GPx).

## 1. Introduction

Parkinson’s disease (PD) is an extrapyramidal syndrome characterized by muscle stiffness, tremors, and bradykinesia that can evolve to loss of motor control, tremors at rest, and postural instability. Other symptoms may be depression and slowness in speech [1,2].

Although the etiology of PD is not entirely clear, the hypothesis of a multifactorial origin of the disease, in which environmental and genetic factors interact, is now accepted [3]. Recent epidemiological studies have shown a correlation between exposure to pollutants, such as pesticides and heavy metals, and the appearance of PD [4]. However, in a significant percentage (about 20%) of patients with positive Parkinson’s familiarity, a genetic cause has been identified [5].

From a biochemical point of view, a reduction in the amount of dopamine, a lower concentration of neuromelanin, a reduction in the activity of the complex I of the mitochondrial respiratory chain, and an increase of reactive oxygen species were found at the substantia nigra *pars compacta* level of parkinsonian subjects [6]. Lewy bodies (insoluble protein aggregates), observed in dopamimergic neurons, may also contribute to the progression of the disease [7]. Furthermore, oxidative stress, dyshomeostasis of metals, mitochondrial dysfunction, and neuroinflammation seem to be involved in the onset and progression of PD [8].

Since there is still no cure for this pathology, many efforts are currently in progress to contrast the onset and/or development of PD. Oxidative stress, due to an imbalance between the production of reactive oxygen species (ROS) and the antioxidant defense system of cells, is currently an important target for the development of new antioxidant molecules. Studies have reported that levels of glutathione (GSH), the main cellular antioxidant, are biochemically altered (decreased) in PD patients [9]. Sulfur compounds, such as cysteine or methionine, are precursors for GSH synthesis. 

Efforts to restore or increase GSH levels were achieved by synthetizing new molecular entities, as reported in Nagasawa et al. [10]. Several peptide transport-mediated prodrugs, obtained linking l-Dopa to GSH, were designed and developed in order to: a) use the GSH uptake transporters that are located on the luminal side of the blood–brain barrier to improve l-Dopa uptake; b) provide targeted delivery of the GSH directly to specific groups of cells, including neurons, where oxidative stress is associated with PD; and c) overcome the pro-oxidant effect associated with L-Dopa (LD) therapy. Another series of prodrug was designed by linking small antioxidant molecules, such as N-acetylcysteine and methionine, to l-Dopa in order to raise the intracellular concentration of GSH, strengthening the natural cellular antioxidant system. Also, cyclic GSH prodrugs were developed to increase GSH intracellular levels and ameliorate the pharmacokinetic profile of the native tripeptide [11].

However, also lower GPx (a Se-dependent enzyme) activity was observed in a PD-affected brain [12]. In this context, selenium and its inorganic and organic derivatives play an important role in the family of antioxidant enzymes, such as GPx. Selenoproteins, containing a residue of selenocysteine in the active site, display a lot of biological functions: immunomodulation, regulation of apoptosis, and control of the redox state of cells [13]. The influence of selenium and its derivatives in several experimental model of PD has been described by several authors; the reduction of the antioxidant brain system caused by selenium deficiency is due to the reduction of GPx activity [14].

**Figure 1:**
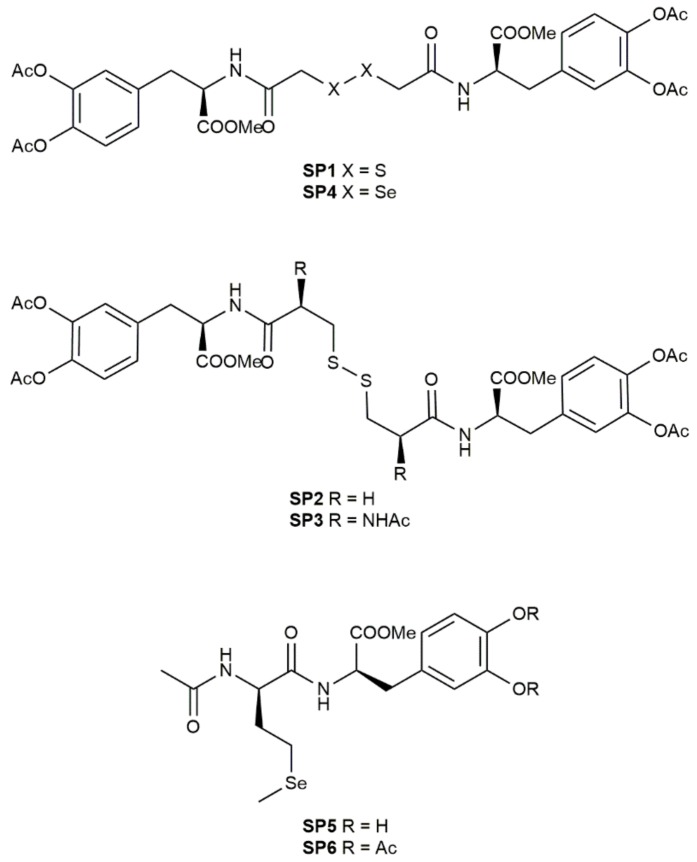
Chemical structures of novel selenyl- and sulfur-L-Dopa derivatives (**SP1-6**).

The aim of this work was to synthetize novel sulfur- and selenyl-l-Dopa (LD) derivatives (**SP1–6**, Figure 1) to increase GSH and LD levels, counteract oxidative stress that lead to loss of dopaminergic neurons, and reinforce the antioxidant defenses of dopaminergic neurons by suppling thiol and selenyl precursors. Notably, selenyl derivatives were designed to restore selenium deficiency that was recently correlated with lower cognitive and impaired motor functions in PD patients [15]. Therefore, the protective role of these novel selenyl derivatives was assayed on SH-SY5Y neuroblastoma cells and compared to the sulfur ones in order to evaluate the potential antioxidant and neuroprotective activities of these novel compounds.

## 2. Materials and Methods

All reagents, unless otherwise stated, were provided by Sigma-Aldrich Co (St. Louis, MO, USA). Thin layer chromatography-polyethylene terephthalate (TLC-PET) foils with fluorescent indicator 254 nm were used. Chromatographic columns were performed on silica gel using column chromatography (Merck 60, 230-400 mesh ASTM silica gel, Merck KGaA, Darmstadt, Germany). Nuclear magnetic resonance (NMR) spectra were recorded with a Varian VXR-300 spectrometer (Varian Medical Systems, Inc., Palo Alto, CA, USA). Mass spectra were obtained using electrospray ionization (ESI) in positive mode with an LCQ (Thermo Finnigan) ion trap mass spectrometer (San Jose, CA, USA) equipped with an electrospray ionization (ESI) source. Analyses indicated by the symbols of the elements or functions were within ± 0.4% of the theoretical values. Purity and chemical structures of **SP1–6** were confirmed using HPLC, and ^1^H-, ^13^C-NMR, and MS spectra (Supplementary Materials **SP1–6**), respectively.

### 2.1. General Procedure for the Synthesis of **SP1–6**

O,O’-Diacetyl-l-dopa-methyl ester hydrochloride (H-LD(Ac)_2_-OMe^.^HCl) (2.1 mmol) was dissolved in dry *N,N*-dimethylformamide (DMF) (15 mL) prior to the addition of triethylamine (TEA) (2.1 mmol) at 0 °C. Then, the suitable sulfur or selenium derivative (0.95 mmol), N,N’-dicyclohexyl carbodiimide (DCC) (2.1 mmol), and HOBt (2.1 mmol) were added and the mixture was stirred for 3 h at 0 °C and then for 15 h at 4 °C. After evaporating the solvent, the crude product was chromatographed on silica gel using the same eluent system reported for TLC for each compound.

*Methyl (2R)-3-[3,4-bis(acetyloxy)phenyl]-2-{2-[({[(2R)-3-[3,4-bis(acetyloxy)phenyl]-1-methoxy-1-oxopropan-2-yl]carbamoyl}methyl)disulfanyl]acetamido}propanoate* (**SP1**). Yield: 54%; Rf = 0.7, CHCl_3_/MeOH (9/1). ^1^H-NMR (300 MHz, CDCl_3_) δ: 2.15 (6H, 2 × s), 3.02-3.10 (2H, m), 3.34–3.42 (2H, m), 3.76 (3H, 1 × s), 4.92 (1H, m), 7.15 (3H, m), 7.09 (1H, m); ^13^C-NMR (75 MHz, CDCl_3_) δ: 20.87, 36.92, 42.36, 52.85, 53.71, 123.70, 124.67, 127.53, 135.11, 144.29, 142.16, 168.45, 168.81, 172.19. Anal. Calcd for C_32_H_36_N_2_O_14_S_2_: C, 52.17; H, 4.93; N, 3.80; O, 30.40; S, 8.70. Found: C, 52.20, H, 4.91, N, 3.78, O, 30.46, S, 8.65. MS (ESI, m/z): 759.09 [M + Na]^+^.

*Methyl (2R)-3-[3,4-bis(acetyloxy)phenyl]-2-{3-[(2-{[(2R)-3-[3,4-bis(acetyloxy)phenyl]-1-methoxy-1-oxopropan-2-yl]carbamoyl}ethyl)disulfanyl]propanamido}propanoate* (**SP2**). Yield: 92%; Rf = 0.8, CHCl_3_/MeOH (9/1). ^1^H-NMR (300 MHz, CDCl_3_) δ: 2.10 (6H, 2 × s), 2.50–2.62 (2H, m), 2.85–2.91 (2H, m), 3.04–3.12 (2H, m), 3.86 (3H, 1 × s), 4.95 (1H, m), 6.67 (1H, d), 7.05–7.11 (3H, m); ^13^C-NMR (75 MHz, CDCl_3_) δ: 20.87, 20.93, 33.82, 35.34, 37.24, 52.78, 53.21, 123.66, 124.74, 127.54, 134.96, 141.29, 142.13, 168.52, 170.96, 172.07. Anal. Calcd for C_34_H_40_N_2_O_14_S_2_: C, 53.39; H, 5.27; N, 3.66; O, 29.29; S, 8.39. Found: C, 53.41; H, 5.29; N, 3.63; O, 29.31; S, 8.36. MS (ESI, m/z): 788.12 [M + Na]^+^.

*Methyl (2R)-3-[3,4-bis(acetyloxy)phenyl]-2-[(2S)-3-{[(2S)-2-{[(2R)-3-[3,4-bis(acetyloxy)phenyl]-1-methoxy-1-oxopropan-2-yl]carbamoyl}-2-acetamidoethyl]disulfanyl}-2-acetamidopropanamido]propanoate* (**SP3**). Yield: 85%; Rf = 0.3, CHCl_3_/MeOH (95/5). ^1^H-NMR (300 MHz, CDCl_3_) δ: 1.98 (3H, s), 2.01 (6H, 2 × s), 2.50–2.60 (2H, m), 3.05–3.15 (2H, m), 3.84 (3H, 1 × s), 4.75 (1H, m), 4.90 (1H, m), 6.45 (1H, d), 6.95-7.16 (4H, m); ^13^C-NMR (75 MHz, CDCl_3_) δ: 19.87, 20.93, 23.91, 33.89, 35.34, 51.99, 52.78, 53.41, 123.76, 124.74, 125.54, 136.96, 140.29, 141.13, 169.52, 171.26, 171.39, 172.07. Anal. Calcd for C_38_H_46_N_4_O_16_S_2_: C, 51.93; H, 5.28; N, 6.37; O, 29.13; S, 7.30. Found: C, 51.90; H, 5.31; N, 6.33; O, 29.15; S, 7.31. MS (ESI, m/z): 902.13 [M + Na]^+^.

*Methyl (2R)-3-[3,4-bis(acetyloxy)phenyl]-2-{2-[({[(2R)-3-[3,4-bis(acetyloxy)phenyl]-1-methoxy-1-oxopropan-2-yl]carbamoyl}methyl)diselanyl]acetamido}propanoate* (**SP4**). Yield: 74%; Rf = 0.7, CHCl_3_/MeOH (9/1). ^1^H-NMR (300 MHz, d_6_-DMSO) δ: 2.15 (6H, 2 × s), 3.02–3.10 (2H, m), 3.34–3.42 (2H, m), 3.76 (3H, 1 × s), 4.92 (1H, m), 7.15 (3H, m), 8.69 (1H, m); ^13^C-NMR (75 MHz, CDCl_3_) δ: 23.26, 26.85, 36.78, 38.11, 52.92, 53.95, 115.59, 117.18, 121.44, 127.45, 143.93, 144.09, 170.04, 171.93, 171.97. Anal. Calcd for C_32_H_36_N_2_O_14_Se_2_: C, 46.28; H, 4.37; N, 3.37; O, 26.97; Se, 19.01. Found: C, 46.30; H, 4.39; N, 3.33; O, 26.95; Se, 19.03. MS (ESI, m/z): 853.76 [M + Na]^+^.

*Methyl (2R)-3-(3,4-dihydroxyphenyl)-2-[(2R)-2-acetamido-4-(methylselanyl)butanamido]-propanoate* (**SP5**). Yield: 56%; Rf = 0.3, CHCl_3_/MeOH (9/1). ^1^H-NMR (300 MHz, CDCl_3_) δ: 1.95 (3H, s), 1.98–2.01 (2H, m), 2.35–2.41 (2H, m), 2.89–2.98 (2H, m), 3.89 (3H, 1 × s), 4.61–4.89 (2H, m), 6.61 (1H, d), 6.59–6.79 (3H, m), 7.10 (1H, m), 8.0 (2H, s); ^13^C-NMR (75 MHz, CDCl_3_) δ: 15.3, 22.0, 31.3, 33.55, 37.9, 51.9, 56.8, 58.2, 116.9, 117.4, 122.03, 130.9, 144.7, 145.6, 172.7, 173.2, 173.9. Anal. Calcd for C_17_H_24_N_2_O_6_Se: C, 47.34; H, 5.61; N, 6.49; O, 22.26; Se, 18.31. Found: C, 47.32; H, 5.68; N, 6.52; O, 22.29; Se, 18.19. MS (ESI, *m/z*): 454.84 [M + Na]^+^.

*Methyl (2R)-3-[3,4-bis(acetyloxy)phenyl]-2-[(2R)-2-acetamido-4-(methylselanyl)butanamido]-propanoate* (**SP6**). Yield: 60%; Rf = 0.4, CHCl_3_/MeOH (95/5). ^1^H-NMR (300 MHz, CDCl_3_) δ: 1.98 (3H, s), 2.0–2.15 (2H, m), 2.10 and 2.17 (6H, 2 × s), 2.45–2.55 (2H, m), 3.06–3.16 (2H, m), 3.84 (3H, 1 × s), 4.55–4.65 (2H, m), 6.14 (1H, d), 6.65 (1H, d), 6.99–7.06 (3H, m); ^13^C-NMR (75 MHz, CDCl_3_) δ: 15.59, 20.88, 23.37, 30.07, 31.29, 37.59, 51.93, 52.77, 54.28, 123.73, 124.76, 127.73, 135.38, 141.25, 142.15, 168.49, 170.45, 170.69, 171.88, 171.89. Anal. Calcd for C_21_H_28_N_2_O_8_Se: C, 48.94; H, 5.48; N, 5.44; O, 24.83; Se, 15.32. Found: C, 48.97; H, 5.45; N, 5.46; O, 24.82; Se, 15.30. MS (ESI, *m/z*): 537.94 [M + Na]^+^.

### 2.2. High Performance Liquid Chromatography (HPLC)-UV Assays

Analytical HPLC measurements were carried out using an HPLC apparatus that consisted of a Waters 600 pump (Waters Corporation, Milford, MA, USA), a 2996 Waters photodiode array detector, and a 7725i Rheodyne manual injector with a 10 μL loop. The mobile phase was a mixture of H_2_O milliQ and MeOH flushing into an RP-C18 column (ODS Hypersil C18 column, 5 µm, 250 x 4.6 mm, 120 Å) with an isocratic flow rate of 0.7 mL/min. The mobile phase was H_2_O/MeOH in the following ratios: 40/60 for **SP1–3**, 20/80 for **SP4**, 60/40 for **SP5**, and 50/50 for **SP6**. The ultraviolet detection was performed at the length of 220 nm.

### 2.3. Lipophilicity

The cl*ogP* values were calculated using ACD LogP software package, version 4.55 (Advanced Chemistry Development Inc., Toronto, Canada). Octanol/water partition coefficients (*logP* o/w) were determined by placing 5 mg of **SP** into 1 mL of anhydrous *n*-octanol and an equal volume of phosphate buffer pH 7.4. After vigorously shaking, the resulting mixture was allowed to stand until the complete separation of the two phases. Finally, each phase was filtered and analyzed using HPLC [16].

### 2.4. Stability in Human Plasma

Human plasma was purchased from 3H Biomedical (Uppsala, Sweden, Europe). **SP** stock solution was added to a mixture of 80% plasma and 20% of 0.02 M phosphate buffer (pH 7.4) at 37 °C to obtain a final drug concentration of 1 mg/mL. Samples of 100 μL were taken at various times, and 200 μL of MeOH containing 0.01 M HCl was used to stop the enzymatic activity. The samples were centrifuged for 5 min at 5000× *g*, and the resultant supernatants were filtered and analyzed using HPLC [17].

### 2.5. Parallel Artificial Membrane Permeability (PAMPA) Method

The blood–brain barrier (BBB)-PAMPA method was used to evaluate the ability of **SP**s to cross the BBB via passive diffusion. The artificial membrane of each microplate was coated with 5 µL of a porcine polar brain lipid extract mixture (purchased from Avantis Polar Lipids, Alabaster, AL, USA). A total of 150 µL of drug solution was put on the membrane (donor plate) while the bottom plate was filled with 300 µL of buffer solution (acceptor plate). The system was incubated for 16 h at room temperature and then the concentration of the drug in the acceptor plate was evaluated using HPLC. The concentration of the drug at the theoretical equilibrium was determined diluting 500 µM of **SP** solution with phosphate buffer pH 7.4 and then analyzed using HPLC [18]. Pe (10^−6^ cm/s) was calculated as the effective permeability coefficient.

### 2.6. SH-SY5Y Cell Culture

Human SH-SY5Y neuroblastoma cells (EGACC, Sigma–Aldrich, Dorset, U.K.) were grown at 37 °C, in a humid 5% CO_2_, in Dulbecco’s Modified Eagle’s medium supplemented with 10% heat-inactivated fetal bovine serum, penicillin (100 U/mL), streptomycin (100 μg/mL), and 1% l-glutammine.

In order to obtain a dopaminergic phenotype, the cells were treated with retinoic acid (RA)/phorbol 12-myristate 13-acetate (PMA) in a medium supplemented with RA (10 μM) for three days; then, the medium was removed and replaced with growth medium containing PMA (80 nM) for three days.

### 2.7. Assessment of Cell Viability

**SP1–6** were dissolved in dimethyl sulfoxide (DMSO). SH-SY5Y cells were plated onto 96-well plates (2700 cell/well). The undifferentiated cells (UC) were grown for 24 h in normal medium and then incubated with seven different compounds at different concentrations (1, 10, and 100 µM). The differentiated cells (DC) were incubated with only two different compounds (**SP6** and LD) at different concentration (1, 10, and 100 µM). After 24 h and 48 h of incubation with or without the compounds, the both sets of cells (UC and DC) were assessed for viability by using a colorimetric assay of 3-[4,5-dimethylthiazol-2-yl]-2,5-diphenyltetrazolium bromide (MTT). The differentiated SH-SY5Y cells were treated with **SP6** or LD compounds at 1 µM for 1 h and then exposed to 25, 50, 75, and 150 µM 6-OHDA. After a further 24 h of incubation, the cultures were assessed for viability using an MTT assay. For experiments with the neurotoxin H_2_O_2_, the differentiated SH-SY5Y cells were pretreated with **SP6** or LD compounds at 1 µM for 24 h and then exposed to 25, 150, and 300 µM H_2_O_2_ for a further 24 h. Cell viability was detected using the MTT assay.

### 2.8. Nitroblue tetrazolium (NBT) Assay

The differentiated SH-SY5Y cells were treated with **SP6** and/or LD compounds at 1 µM for 24 h, and then 10^6^ cells were harvested, centrifuged for 5 min at 1200 rpm, and resuspended in 1 mL 0.9% NaCl, in which NBT (nitro blue tetrazolium chloride, 1 mg/mL) was dissolved. The cells were incubated for 3 h at 37 °C, centrifuged (10 min at 1200 rpm), resuspended in 1 mL dimethyl sulfoxide, and left for 20 min at 37 °C. Then, cells were plated in a 96 well plate (2 x 10^5^ cell/well) and read at 550 nm using a scanning multi-well spectrophotometer (Cary50MPR, Varian).

### 2.9. Intracellular Reactive Oxygen Species Measurement

SHSY-5Y human neuroblastoma cells were plated (2700 cells/well) into special optics 96-well plates (Corning-Costar, New York, USA) and the cells were treated with retinoic acid (RA)/phorbol 12-myristate 13-acetate (PMA) in order to obtain a dopaminergic phenotype. The differentiated cells were treated with **SP1–6** at 1 µM for 24 h and then washed with an imaging buffer (125 mM NaCl, 5 mM KCl, 1.2 mM MgSO_4_, 5 mM glucose, 25 mM HEPES, and 2 mM CaCl_2_). A concentration of 10 µM of 2′,7′-dichlorofluorescin diacetate (H_2_DCFDA, Molecular probes, Life Technologies REF D399) was added and the plates were incubated at 37 °C for 30 min. After the incubation, cells were washed with an imaging buffer and treated with 25 µM H_2_O_2_ for an immediate fluorescence measurement.

Plates were read every 50 s from 0 to 5 min for kinetic data analysis using a microplate reader (Synergy H1, BioTek), with excitation and emission wavelengths at 490 nm and 520 nm, respectively, and analyzed by Gen 5 version 2.08 (BioTek).

### 2.10. Statistical Analysis

The results of biological analysis are presented as the mean ± standard deviation of three repetitions. The statistical analysis (unpaired *t*-tests) was carried out using the GraphPad Prism Software, version 7 (GraphPad Software, La Jolla, California, USA), comparing treated cells respect to the control.

## 3. Results

**Scheme 1:**
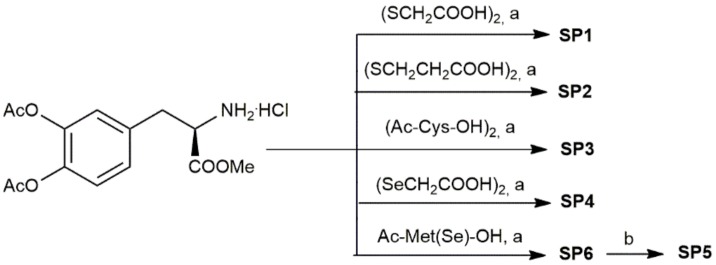
Reagents and conditions: N,N’-dicyclohexyl carbodiimide (DCC), 1- hydroxybenzotriazole (HOBt), triethylamine (TEA), *N,N*-dimethylformamide (DMF), 3 h at 0 °C then 15 h at 4 °C; (**b**) NaHCO_3_, 80 min at room temperature.

A series of sulfur and selenyl compounds containing LD were successfully synthesized according to the reaction Scheme 1. The synthesis of compounds **SP1–6** was performed using N,N’-dicyclohexylcarbodiimide (DCC) as a coupling reagent, in the presence of hydroxybenzotriazole (HOBt), to avoid racemization of the chiral centers, triethylamine (TEA), and N,N-dimethylformamide (DMF). **SP6** was also subjected to the removal of catechol acetyl groups by using mild basic conditions to give **SP5**. All compounds were obtained in satisfactory yields (>50%) and fully characterized using NMR and MS spectra (Supplementary Materials).

**SP1–6** were also assayed for the evaluation of physico-chemical properties, such as lipophilicity (clogP, logP), ability to cross the blood–brain barrier (BBB), and plasma stability. Results showed that the most lipophilic derivative was **SP6** (logP = 1.435) suggesting that it could easily pass through the biological membranes compared to LD (logP = −2.457) (Table 1). These data were also confirmed using a blood–brain barrier permeability assay (BBB-PAMPA) (Table 2). The BBB-PAMPA assay was based on a 2% *w*/*v n*-dodecane solution of porcine brain tissue extract and successfully differentiated CNS^+^ from CNS^−^ compounds [19]. Results showed that **SP6** and **SP3** possess the capacity to cross the BBB compared to other derivatives in accordance with the logP values. **SP5**, containing the free catechol group with respect to **SP6**, resulted in being unable to completely cross the BBB.

Plasma stability studies were also performed to evaluate the stability of **SP1–6** in the presence of plasma enzymes (Table 3). Data revealed that, except for **SP4**, all **SP**s were stable in plasma; notably, **SP5** and **SP6** displayed a half-life that was longer than 2 h, suggesting that they were not sensible to proteases degradation.

**Figure 2:**
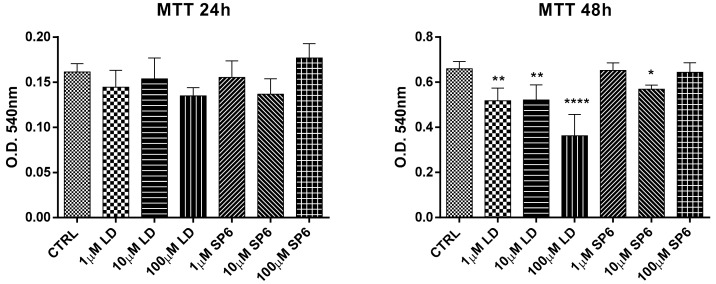
Dose-response effects of LD and **SP6** in undifferentiated SH-SY5Y human neuroblastoma cells. MTT reduction assay in undifferentiated SH-SY5Y human neuroblastoma cells in the presence of LD and **SP6**. The cells were incubated for 24 or 48 h with increasing concentrations (1, 10, and 100 µM) of the compounds. After this period, cell viability was quantified by measuring MTT reduction. CTRL: control without compounds. The means ± scanning electron microscopy (SEM) were derived from three different experiments (each with *n* = 16; **** *p* < 0.0001; ** 0.0005 < *p* < 0.001; * 0.001 < *p* < 0.05; n.s., *p* > 0.05).

Before performing the neuroprotective studies, the cytotoxic action of LD (l-*p*-3,4-dihydroxyphenylalanine hydrochloride) and several synthetized compounds (**SP1–6**) at 1, 10, and 100 µM concentrations at 24 and 48 h was evaluated on SY-SH5Y undifferentiated cells. Based on this preliminary evaluation, reported in the Supplementary Material (Figure S1), **SP6** was selected for the following experiments. Therefore, the antioxidant and neuroprotective capacities of **SP6** against oxidative stress were assayed by using the human SH-SY5Y neuroblastoma cell line. As shown in Figure 2, after 24 h of incubation, both LD and **SP6** did not show cytotoxic effects for all tested concentrations, while at 48 h, LD negatively interfered with cellular vitality by showing a toxic effect at 100 µM.

**Figure 3:**
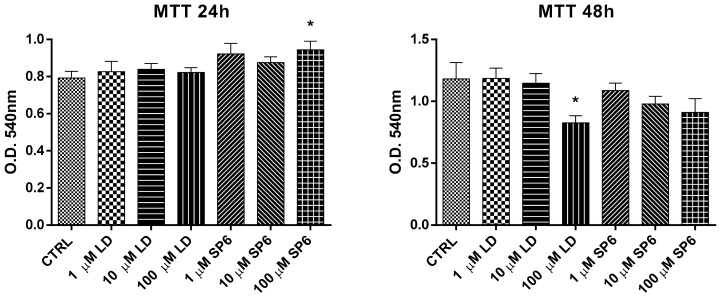
Dose-response effects of LD and **SP6** in differentiated SH-SY5Y human neuroblastoma cells. MTT reduction assay in differentiated SH-SY5Y human neuroblastoma cells in the presence of LD and **SP6**. The cells were incubated for 24 or 48 h with increasing concentrations (1, 10, and 100 µM) of the compounds. After this period, cell viability was quantified by measuring the MTT reduction. CTRL: control without compounds. The means ± SEM were derived from three different experiments (each with *n* = 16; * 0.001 < *p* < 0.05; n.s., *p* > 0.05).

Similar behavior was also observed in SY-SH5Y dopaminergic differentiated cells at 24 and 48 h with a detrimental effect of LD (100 µM) at 48 h (Figure 3).

**Figure 4:**
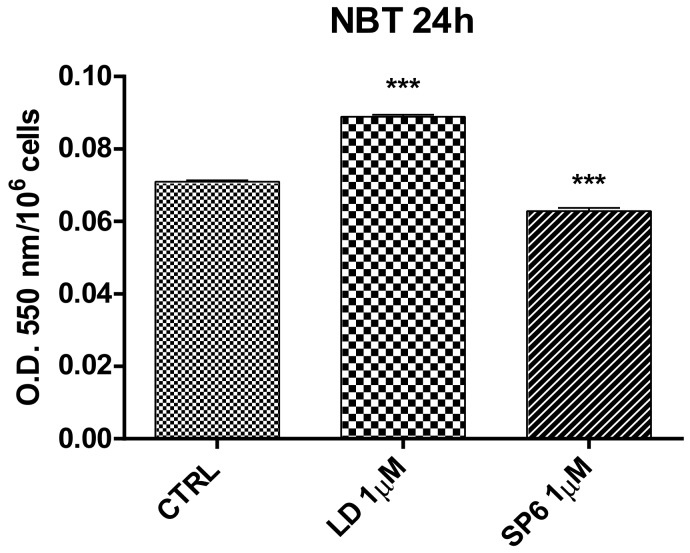
Antioxidant activity of LD and **SP6** against oxidative stress measured using the NBT test with differentiated SH-SY5Y neuroblastoma cells. Results were obtained after 24 h of incubation with LD and **SP6** (1 µM). CTRL: control without compounds. Mean values ± SEM were derived from three different experiments (each with *n* = 10; *** *p* < 0.0001).

For this reason, in all subsequent experiments, LD and **SP6** were used at 1 µM. The antioxidant activity was evaluated using an NBT assay. Results showed that **SP6** significantly reduced the superoxide anion level, while LD even seemed to increase it (Figure 4).

**Figure 5:**
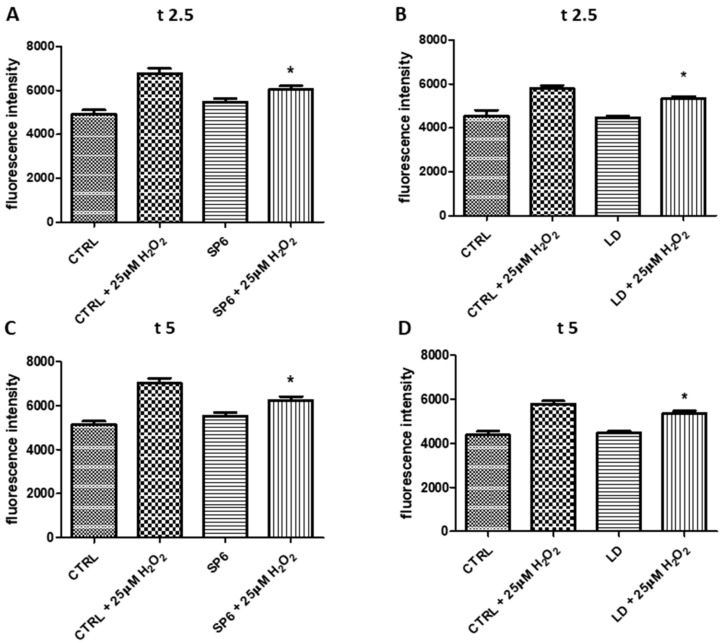
Measurement of the intracellular reactive oxygen species (ROS). The differentiated SY-SH5Y cells incubated with 1 µM **SP6** (panels **A** and **C**) or LD (panels **B** and **D**) for 24 h, were treated with 25 µM H_2_O_2_ for 5 min. Figure 7 reported the fluorescence intensities at two points (t_2.5_ and t_5_) during the assays. The means ± SEM derived from two different experiments (each with *n* = 8; * 0.001 < *p* < 0.05; n.s., *p* > 0.05).

The antioxidant activity of **SP1-6** was also evaluated using an H_2_DCFDA assay (Figure 5 and Figure S2). During the assays (in Figure 5, times are reported as 2.5 and 5 min), both **SP6** and LD showed a significant antioxidant activity with respect to the CTRL in the presence of 25 µM H_2_O_2_ (CTRL + 25 µM H_2_O_2_).

**Figure 6:**
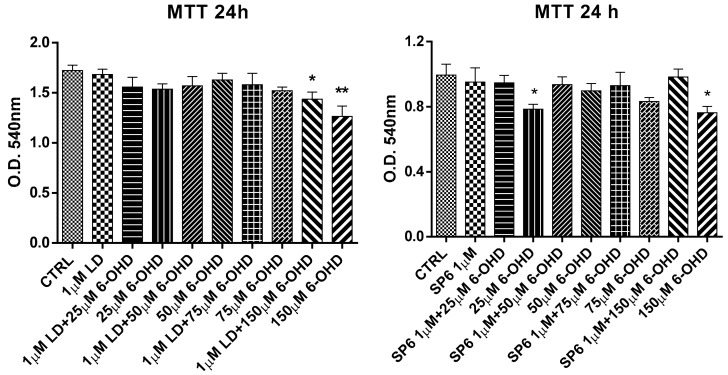
Neuroprotective effect of LD and **SP6** against 6-OHDA in RA/PMA-differentiated SH-SY5Y human neuroblastoma cells. The MTT reduction assay in RA/PMA-differentiated and 6-OHDA-lesioned SH-SY5Y human neuroblastoma cells in the presence of LD and **SP6**. The cells were incubated with the compounds (1 µM) 1 h before and during a 24 h incubation period with increasing concentrations (25, 50, 75, and 150 µM) of 6-OHDA. After this period, cell viability was quantified by measuring the MTT reduction. CTRL: control without compounds. The means ± SEM derived from three different experiments (each with *n* = 16; ** 0.0005 < *p* < 0.001; * 0.001 < *p* < 0.05; n.s., *p* > 0.05).

To investigate the neuroprotective role of our compounds, the SH-SY5Y neuroblastoma cells were differentiated toward the DAergic phenotype using retinoic acid/phorbol myristyl acetate (RA/PMA). The treatment of differentiated cells with LD and **SP6** for 1 h was followed by the exposure to increasing concentrations of 6-OHDA (25, 50, 75, 150 µM). After a further 24 h of incubation, the cultures were assessed for viability using an MTT assay (Figure 6). Results showed that **SP6** demonstrated a protective action against the toxin at all tested concentrations, while LD was able to partially counteract the toxic effect of 150 µM 6-OHDA.

**Figure 7:**
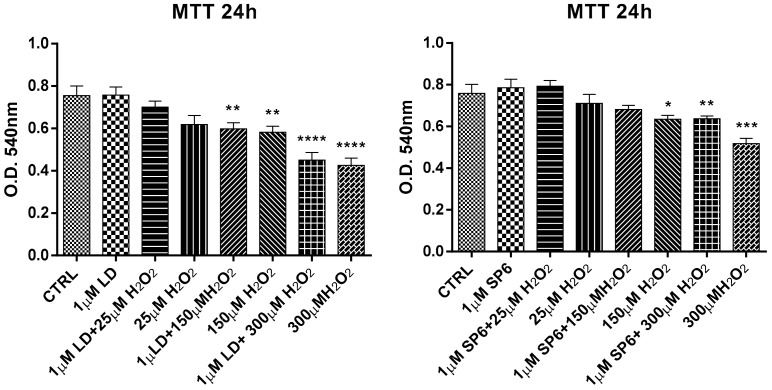
Neuroprotective effect of LD and **SP6** against H_2_O_2_ in RA/PMA-differentiated SH-SY5Y human neuroblastoma cells. The MTT reduction assay in differentiated and H_2_O_2_-lesioned SH-SY5Y human neuroblastoma cells in the presence of LD and **SP6**. The cells were incubated with the compounds (1 µM) 24 h before and during a 24 h incubation period with increasing concentrations (25, 150, or 300 µM) of H_2_O_2_. After this period, cell viability was quantified by measuring the MTT reduction. CTRL: control without compounds. The means ± SEM derived from three different experiments (each with *n* = 16; **** *p* < 0.0001; *** 0.0001 < *p* < 0.0005; ** 0.0005 < *p* < 0.001; * 0.001 < *p* < 0.05; n.s., *p* > 0.05).

Furthermore, the differentiated cells with RA/PMA were lesioned with increasing concentrations (25, 150, 300 µM) of H_2_O_2_ (Figure 7). Data showed that **SP6** was effective in countering the toxic effect of 150 µM H_2_O_2_, and partially countered 300 µM H_2_O_2_, while LD was ineffective at both 150 and 300 µM H_2_O_2_.

## 4. Discussion

It is now overt that enhanced oxidative stress is an important pathogenetic mediator in Parkinson’s disease. Usually, PD patients are treated with LD, but this drug is not able to stop the progression of the disease [20]. It was even considered that LD treatment may exacerbate the progress of degeneration of dopaminergic neurons in PD patients. Indeed, reactive oxygen species can potentially be generated by the metabolism and/or the autoxidation of LD itself [21]. Therefore, it is possible that LD treatment may increase oxidative stress toward the nigrostriatal dopaminergic neurons, which have already suffered from enhanced oxidative stress. For this reason, in this study we used an SH-SY5Y human neuroblastoma cells line, differentiated toward a dopaminergic phenotype, which is a suitable model for studying the neurotoxic effects of agents like H_2_O_2_ and 6-OHDA, and understanding the mechanisms of neurodegenerative diseases [22]. The well-known behavior of LD could explain on the one hand the inhibiting effect of cell proliferation, which is evident at 48 h at all LD used concentrations, and on the other hand, an increase in superoxide anion levels, even at 1 µM. On the contrary, **SP6** had a significant protective effect against the neurotoxic action of 6-OHDA and H_2_O_2_, proving to be an effective antioxidant and protective compound. Moreover, **SP6** was able to reduce ROS levels following the addition of H_2_O_2_.

In fact, **SP6**, counteracting these two deleterious toxins, could increase the antioxidant system of dopaminergic cells, and at the same time, supply selenium, whose deficiency in PD patients is due to the reduction of GPx activity. Other selenium-dependent enzymes, such as thioredoxin reductase 1 and 2, selenoprotein P, and some isoforms of methionine sulfoxide reductases are involved in the mechanisms of cellular protection against oxidative stress. Moreover, decreased GSH levels were reported in the substantia nigra *pars compacta* and striatum of PD patients [10].

**SP6** may release seleno-l-methionine, after hydrolysis of the amide bond, that is more reactive with oxidants compared to a sulfur analog (RSH, pKa about 8.7), because at physiological pH, its nucleophilicity (RSe^−^, pKa 5.2) is higher compared to the corresponding thiolate [23].

Therefore, the incorporation of selenium into **SP6** could provide protection against 6-OHDA and H_2_O_2_-induced dopaminergic neurotoxicity by modulating GPx activity. Moreover, the physico-chemical characterization of **SP6** revealed that it was endowed with the suitable properties that allow it to be resistant to the enzymatic hydrolyses by plasma esterases and pass through the BBB. The lipophilic nature and low molecular weight (MW = 515.42) of **SP6** could facilitate the crossing of biological membranes via passive diffusion compared to other derivatives (MW > 740), thus selectively reaching the target tissues.

In conclusion, selenyl-LD derivatives have great potential for developing important pharmacological approaches for neurodegenerative diseases, such as PD. Further studies will help define the exact antioxidant and neuroprotective mechanism of **SP6** and determine whether the neuroprotective action is mediated/modulated by GPx or by enzymatic pathways.

## Figures and Tables

**Table 1:** Physico-chemical properties of **SP1-6**.

	L-Dopa	SP1	SP2	SP3	SP4	SP5	SP6
clogP ^1^	−2.82	−0.126	0.620	0.458	−1.358	0.167	0.678
logP	−2.457 (±0.119)	−0.163 (±0.02)	0.828 (±0.10)	1.069 (±0.003)	−1.172 (±0.003)	0.510 (±0.025)	1.435 (±0.037)

^1^ Values are calculated with ACD LogP software package, version 4.55.

**Table 2:** Parallel artificial membrane permeability blood brain barrier (PAMPA-BBB) data of **SP1–6.**

	LD	SP1	SP2	SP3	SP4	SP5	SP6
Pe (10^−6^ cm/s)	0.75 (±1.67)	1.15 (±0.12)	1.82 (±0.18)	3.1 (±1.3)	0.94 (±0.05)	2.3 (±0.83)	4.5 (±0.88)
Classification ^1^	CNS^−^	CNS^−^	CNS^−^	CNS^+/−^	CNS^+^	CNS^−^	CNS^+/−^

^1^ CNS^+^ (indicative of high BBB permeation): Pe (10^−6^ cm/s) > 4; CNS^+/-^ (indicative of uncertain BBB permeation): Pe (10^−6^ cm/s) from 4 to 2; CNS^−^ (indicative of low BBB permeation): Pe (10^−6^ cm/s) < 2.

**Table 3:** Plasma stability studies of **SP1–6.**

	SP1	SP2	SP3	SP4	SP5	SP6
*t*_1/2_ (min)	28.8 (±0.932)	66.2 (±4.455)	50.2 (±10.061)	Immediate hydrolysis	126.0 (±3.551)	173.4 (±6.130)
K_obs_ (min^−1^)	0.024 (±0.001)	0.010 (±0.001)	0.014 (±0.003)		0.005 (±0.0001)	0.004 (±0.0001)

## Supplementary Materials

The following are available online at https://www.mdpi.com/2218-273X/9/6/239/s1, Figure S1: Dose-response effects of LD and **SP1**–**5** in undifferentiated SH-SY5Y human neuroblastoma cells. Figure S2: Measurement of intracellular reactive oxygen species (ROS). NMR and MS spectra of **SP1**–**6** are reported in the Supplementary Materials.
