# Supplementary material for: Synthesis and Biological Evaluation of Novel Selenyl and Sulfur-l-Dopa Derivatives as Potential Anti-Parkinson’s Disease Agents
Source: Biomolecules. 2019 Jun 18;9(6):239. doi: 10.3390/biom9060239 (PMC6628379; doi:10.3390/biom9060239)
Supplement: Supplementary file 1 [file biomolecules-09-00239-s001.pdf]

Article

Synthesis and biological evaluation of novel selenyl- and sulfur-L-Dopa derivatives as potential anti-Parkinson compounds

Antonio Di Stefano [1], Lisa Marinelli[1], Piera Eusepi[1], Michele Ciulla[1], Stefania Fulle[2], Ester Sara Di Filippo[2], Laura Magliulo[2], Giuseppe Di Biase[1], and Ivana Cacciatore[1,*]

[1] Department of Pharmacy, University "G. d'Annunzio" Chieti-Pescara, Chieti, Italy
[2] Department of Neuroscience, Imaging and Clinical Sciences, University "G. d'Annunzio" Chieti-Pescara, Chieti, Italy
* Correspondence: ivana.cacciatore@unich.it; Tel.: +39-0871-355-4475

Supplementary material

^{1}H-, ^{13}C-NMR and MS spectra of SP1
^{1}H-, ^{13}C-NMR and MS spectra of SP2
^{1}H-, ^{13}C-NMR and MS spectra of SP3
^{1}H-, ^{13}C-NMR and MS spectra of SP4
^{1}H-, ^{13}C-NMR and MS spectra of SP5
^{1}H-, ^{13}C-NMR and MS spectra of SP6
MTT assay for SP1–5
Antioxidant test for SP1–5

SP1

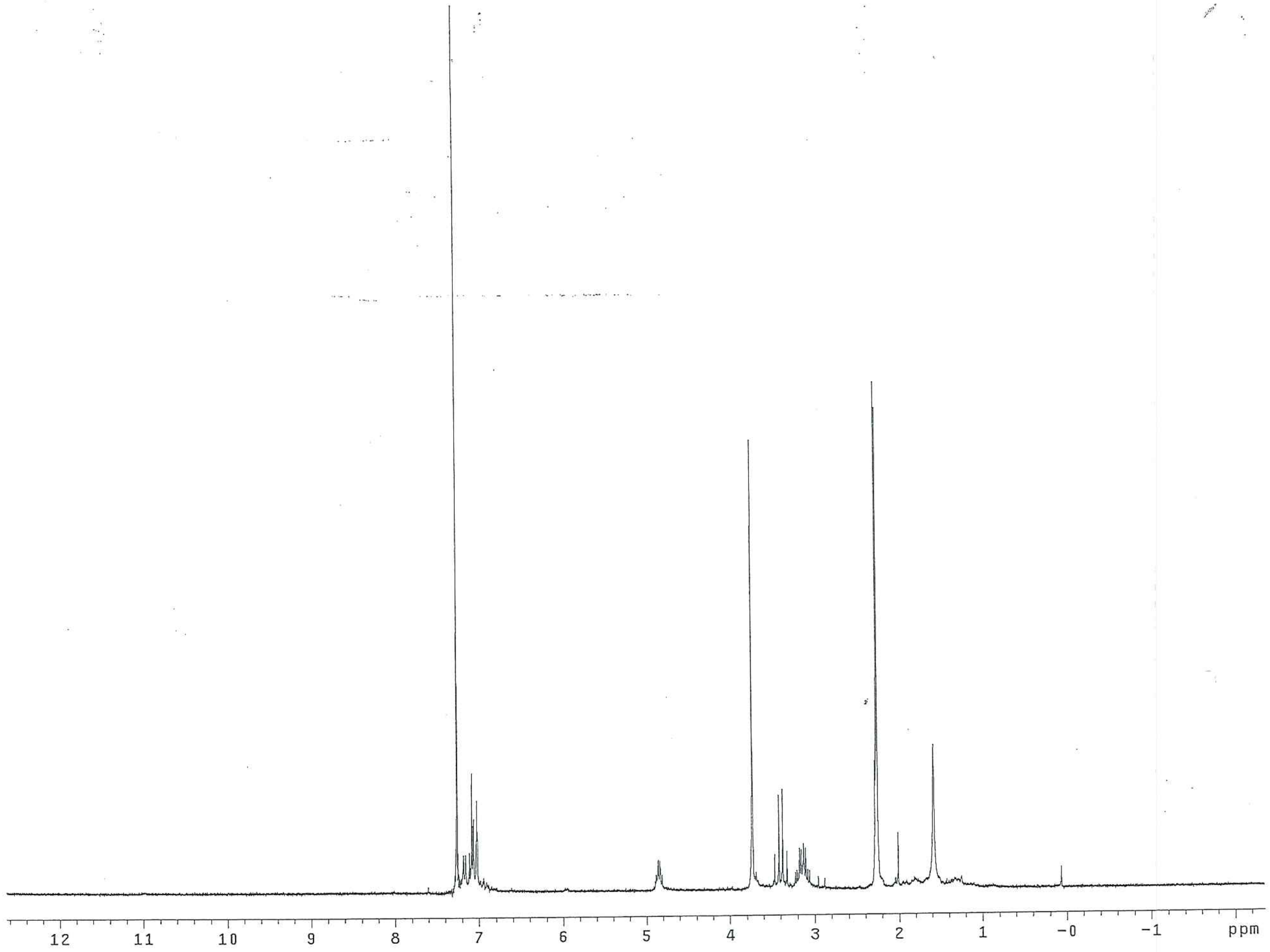

SP1

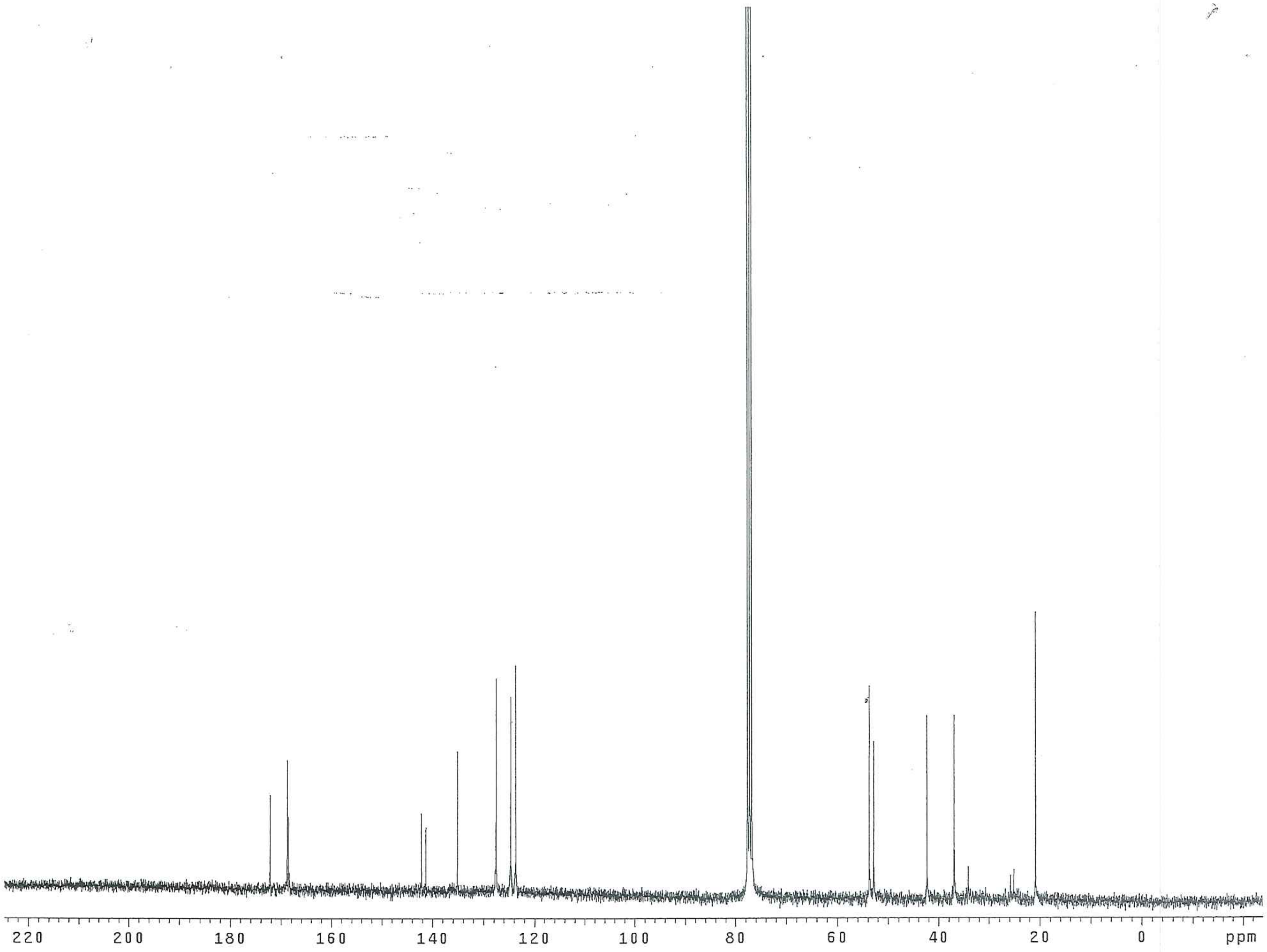

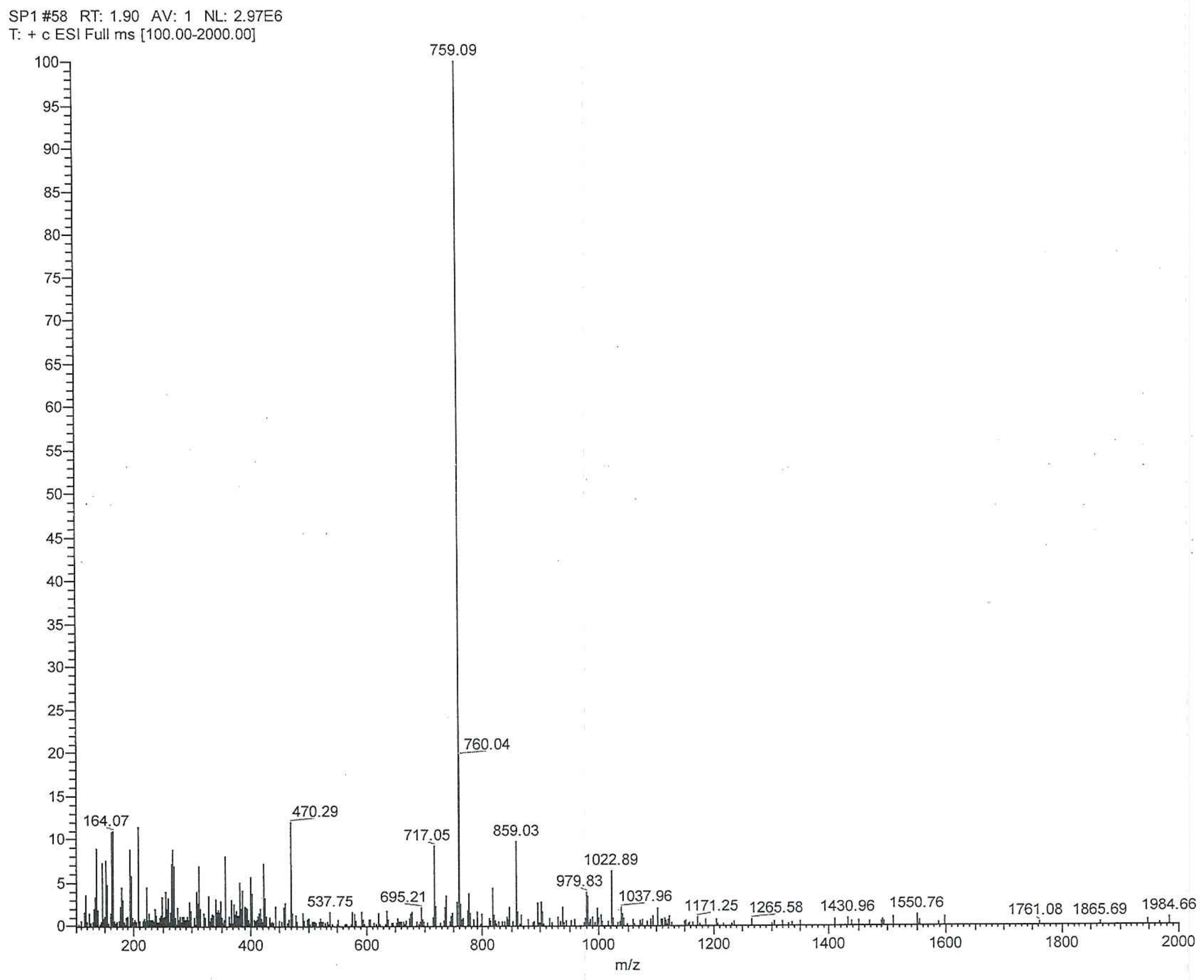
SP1 #58 RT: 1.90 AV: 1 NL: 2.97E6
T: + c ESI Full ms [100.00-2000.00]
759.09
760.04
164.07
470.29
717.05
859.03
1022.89
979.83
537.75
695.21
1037.96
1171.25
1265.58
1430.96
1550.76
1761.08
1865.69
1984.66
m/z

^1H-NMR ($CDCl_3$)

SP2

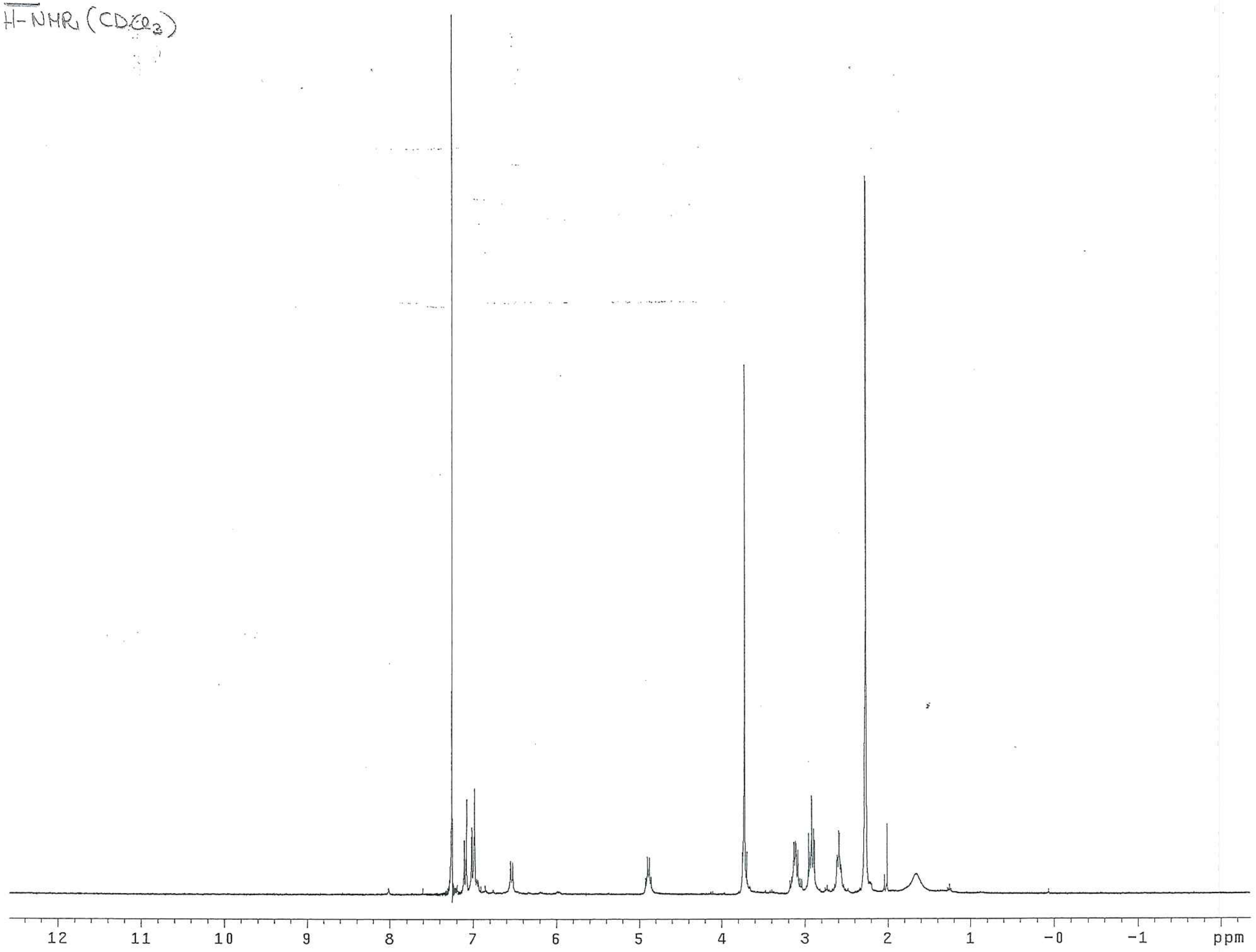

SP2

13C OBSERVE

exp1 std13c

SAMPLE		DEC. & VT	
date	Nov 28 2007	dfrq	300.199
solvent	CDCl3	dn	H1
file	exp	dpwr	41
ACQUISITION		dof	0
sfrq	75.492	dm	yyy
tn	C13	dmm	w
at	1.815	dmf	11400
np	68106	PROCESSING	
sw	18761.7	lb	1.00
fb	10400	wtfile	
bs	16	proc	ft
tpwr	62	fn	not used
pw	8.7		
d1	0	werr	
tof	0	wexp	
nt	1e+08	wbs	
ct	35264	wnt	
alock	s		
gain	not used		

FLAGS	
il	n
in	n
dp	y

DISPLAY	
sp	-1804.1
wp	18761.7
vs	217
sc	0
wc	250
hzmm	20.12
is	500.00
rfl	1804.1
rfp	0
th	64
ins	100.000
nm no ph	

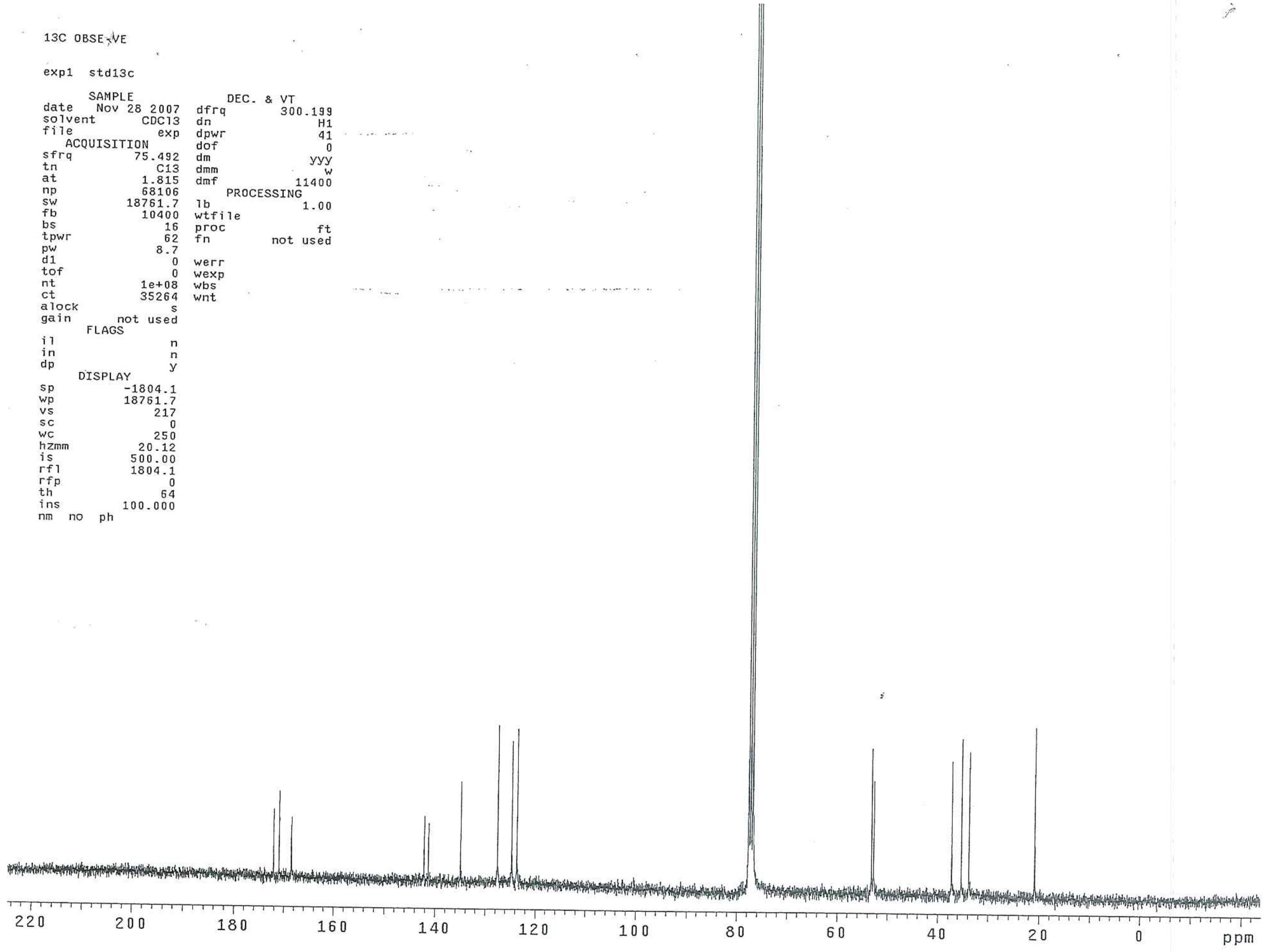

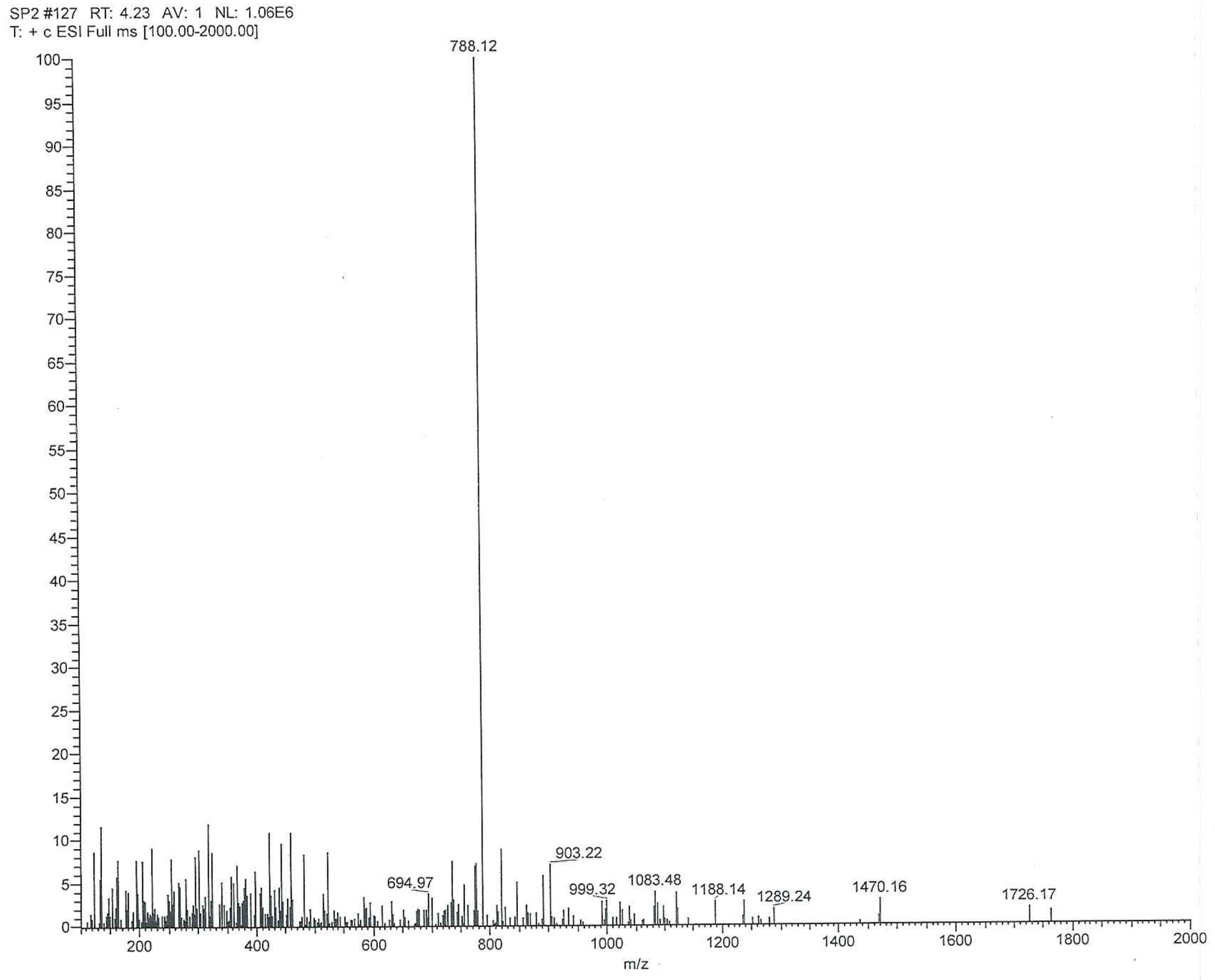
SP2 #127 RT: 4.23 AV: 1 NL: 1.06E6
T: + c ESI Full ms [100.00-2000.00]
788.12
903.22
694.97
999.32
1083.48
1188.14
1289.24
1470.16
1726.17
m/z

1H-NMR ($CDCl_3$)

SP3

STANDARD 1H OBSERVE

exp1 std1h

SAMPLE		DEC. & VT	
date	Mar 28 2007	dfrq	300.059
solvent	CDCl3	dn	H1
file	exp	dpwr	41
ACQUISITION		dof	0
sfrq	300.059	dm	nnn
tn	H1	dmm	c
at	1.998	dmf	11400
np	17984	PROCESSING	
sw	4500.5	wtfile	
fb	2600	proc	ft
bs	16	fn	not used
tpwr	57		
pw	7.0	werr	
d1	1.000	wexp	
tof	0	wbs	
nt	32	wnt	
ct	32		
alock	n		
gain	20		
FLAGS			
il	n		
in	n		
dp	y		
DISPLAY			
sp	-804.1		
wp	4500.5		
vs	214		
sc	0		
wc	250		
hzmm	18.00		
is	823.53		
rfl	804.1		
rfp	0		
th	20		
ins	1.000		
nm cdc ph			

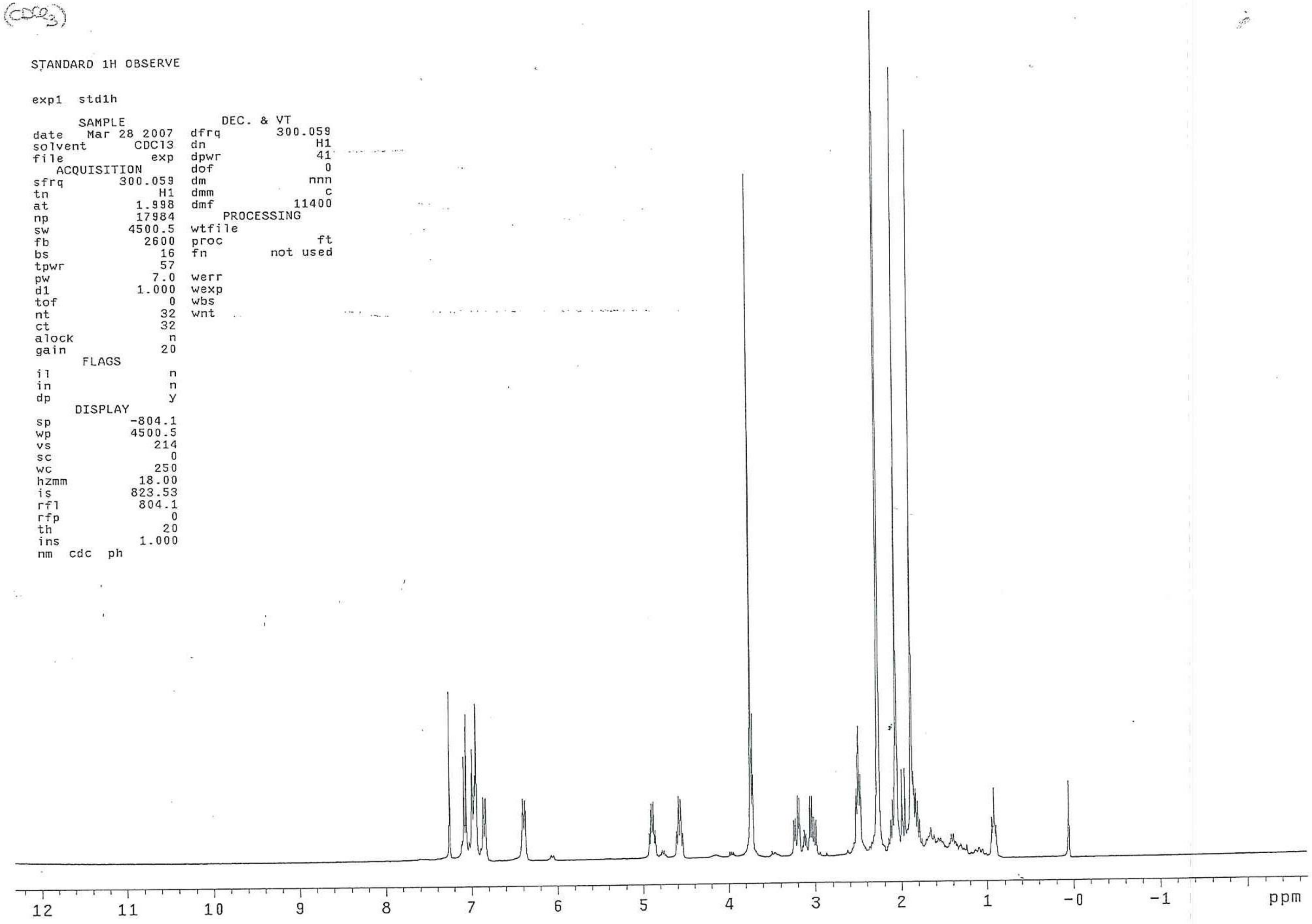

SP3

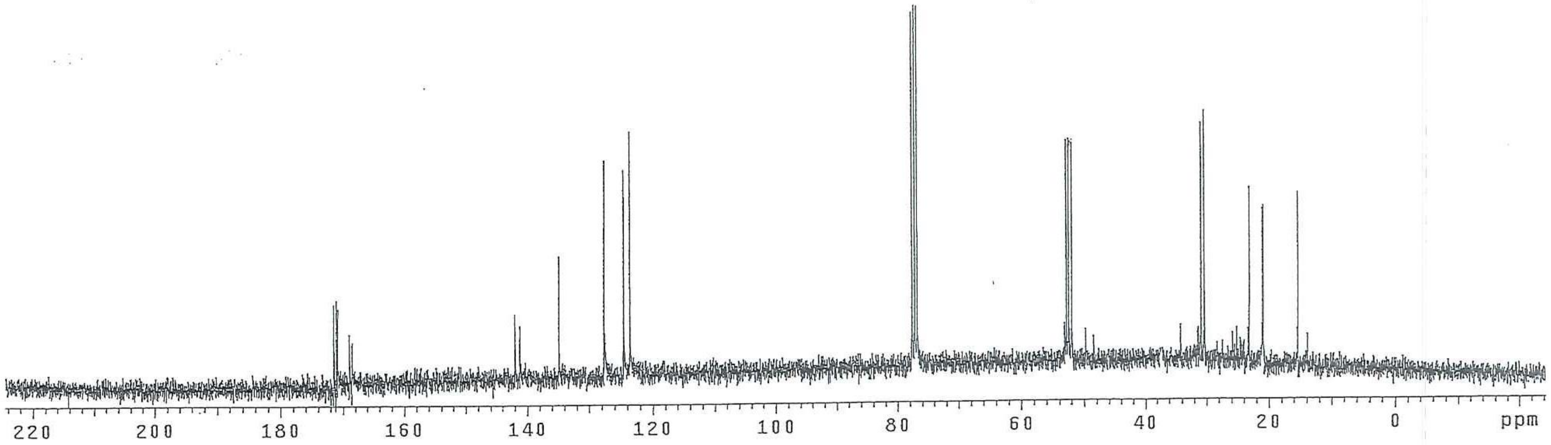

SP3 #105 RT: 3.47 AV: 1 NL: 1.44E6
T: + c ESI Full ms [100.00-2000.00]

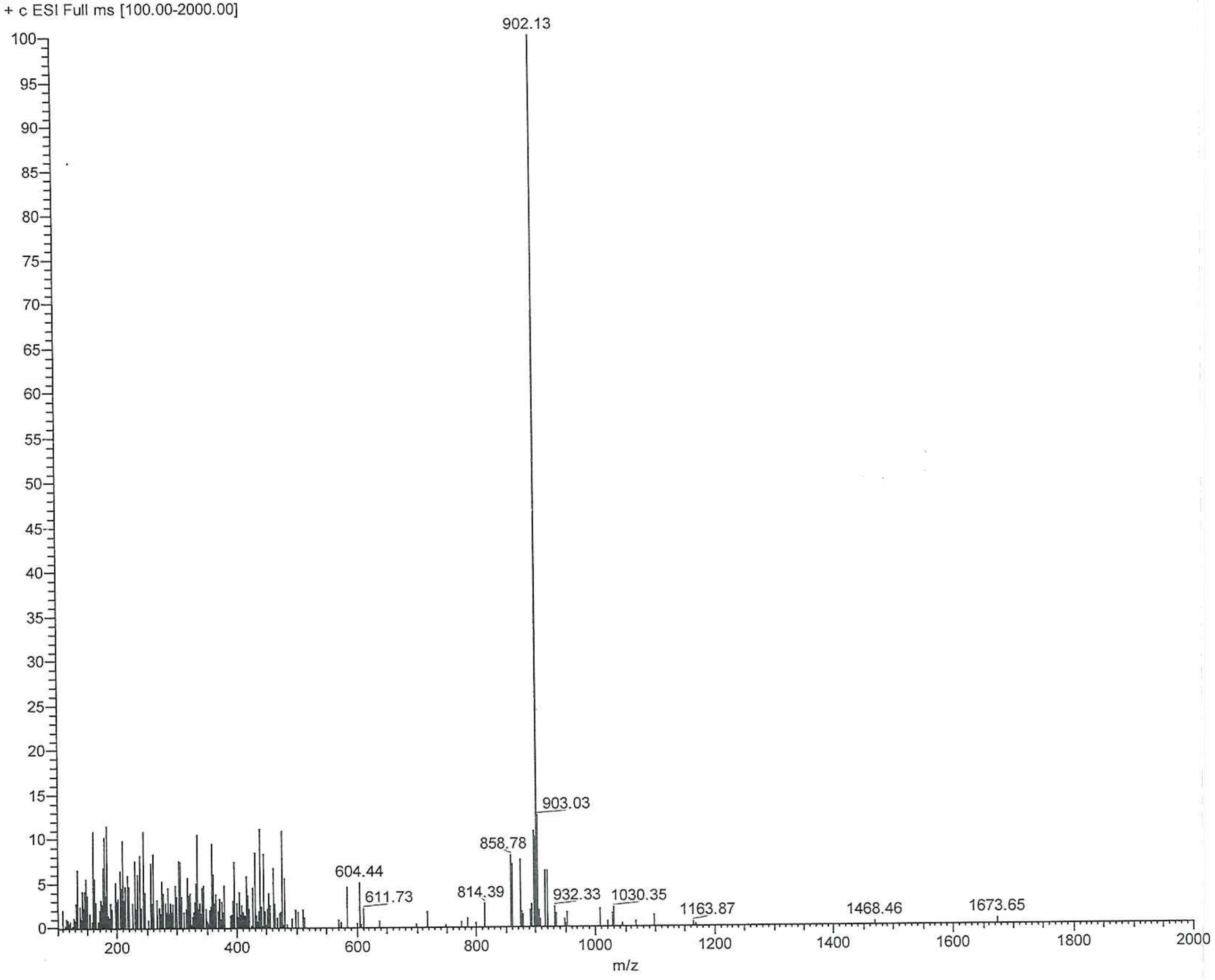

SP4

STANDARD 1H OBSERVE

```
exp1  std1h

      SAMPLE                DEC. & VT
date    Jan 28 2008   dfrq        300.201
solvent        DMSO   dn               H1
file            exp   dpwr             41
   ACQUISITION        dof               0
sfrq        300.201   dm              nnn
tn               H1   dmm               c
at            1.998   dmf           11400
np            17984       PROCESSING
sw           4500.5   wtfile
fb             2600   proc             ft
bs               16   fn         not used
tpwr             57
pw              7.0   werr
d1            1.000   wexp
tof               0   wbs
nt               32   wnt
ct               32
alock             n
gain             20
      FLAGS
il                n
in                n
dp                y
     DISPLAY
sp           -706.4
wp           4500.5
vs              214
sc                0
wc              250
hzmm           4.00
is           500.00
rfl           706.4
rfp               0
th               20
ins         100.000
nm  cdc  ph
```

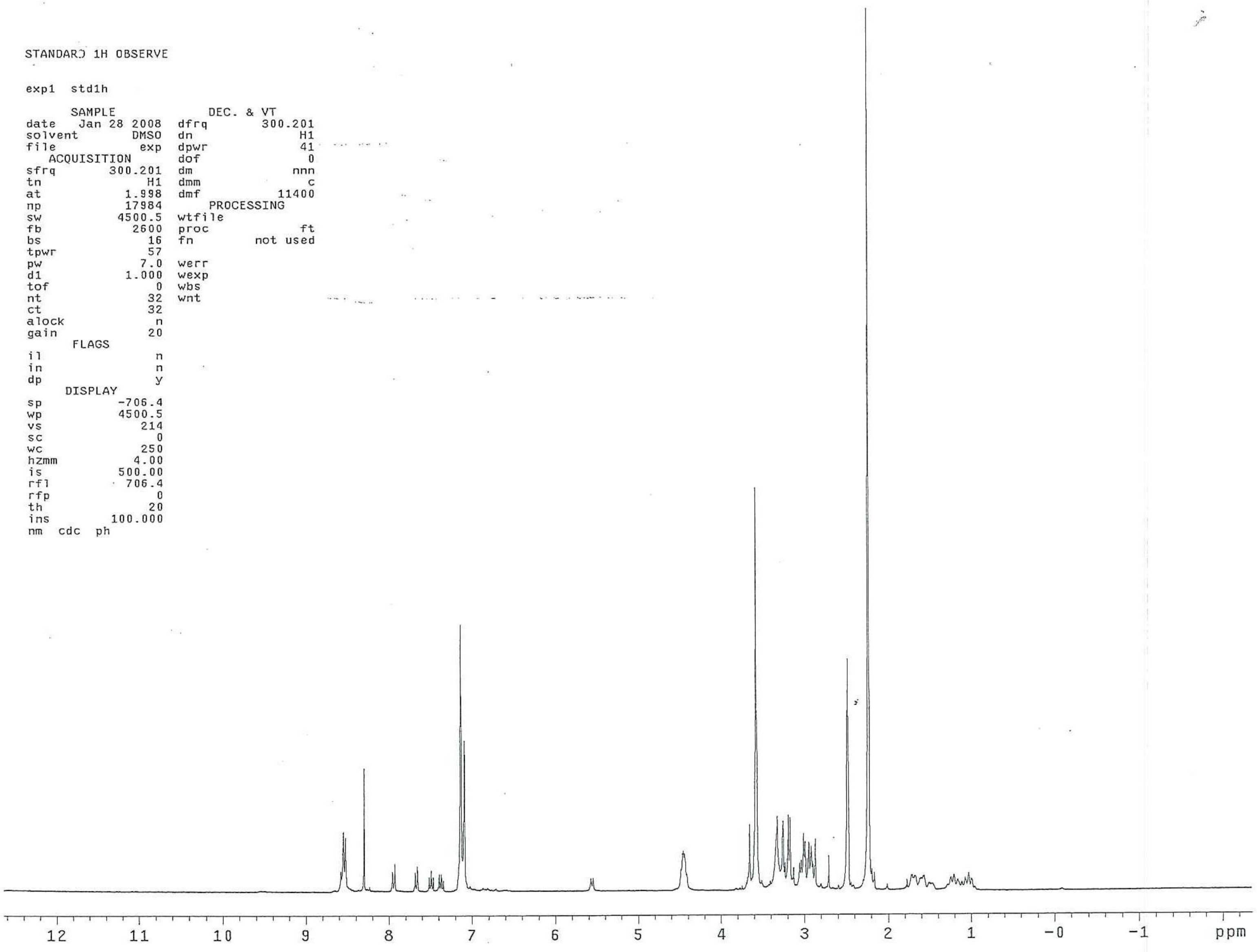

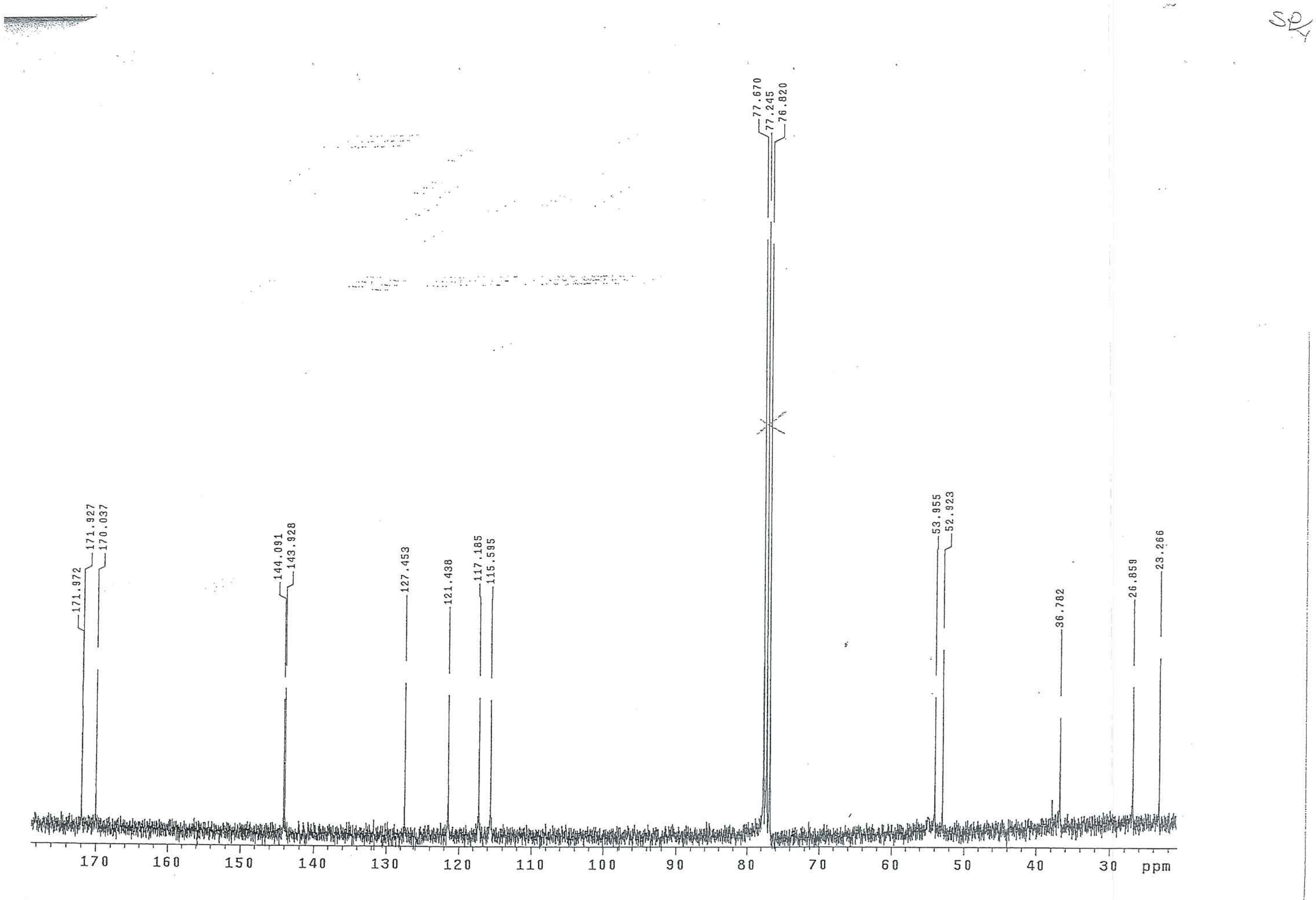

SP4
171.972
171.927
170.037
144.091
143.928
127.453
121.438
117.185
115.595
77.670
77.245
76.820
53.955
52.923
36.782
26.859
23.266
170 160 150 140 130 120 110 100 90 80 70 60 50 40 30 ppm

SP4 #60 RT: 1.97 AV: 1 NL: 2.23E6
T: + c ESI Full ms [100.00-2000.00]

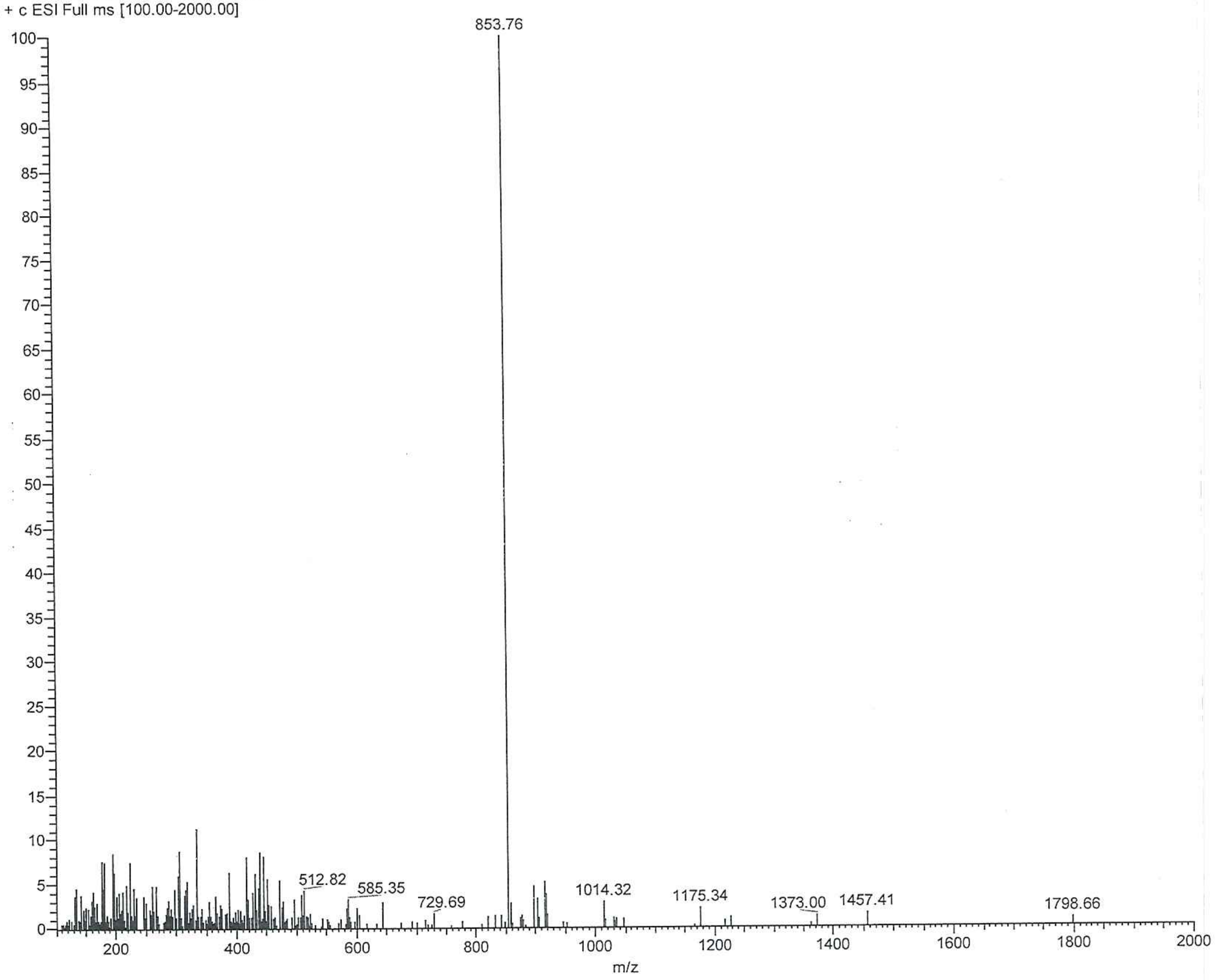

SP5

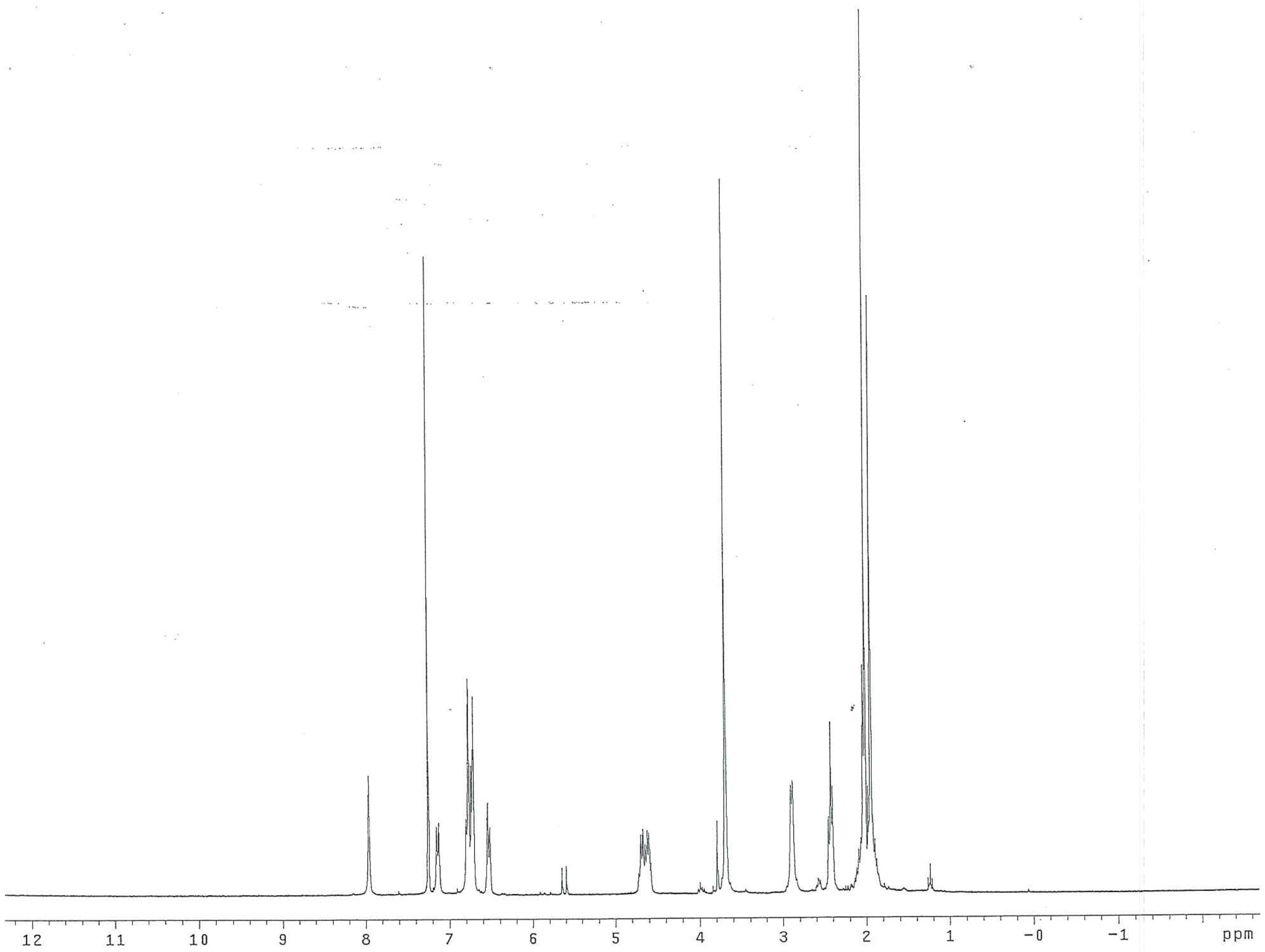

SP5

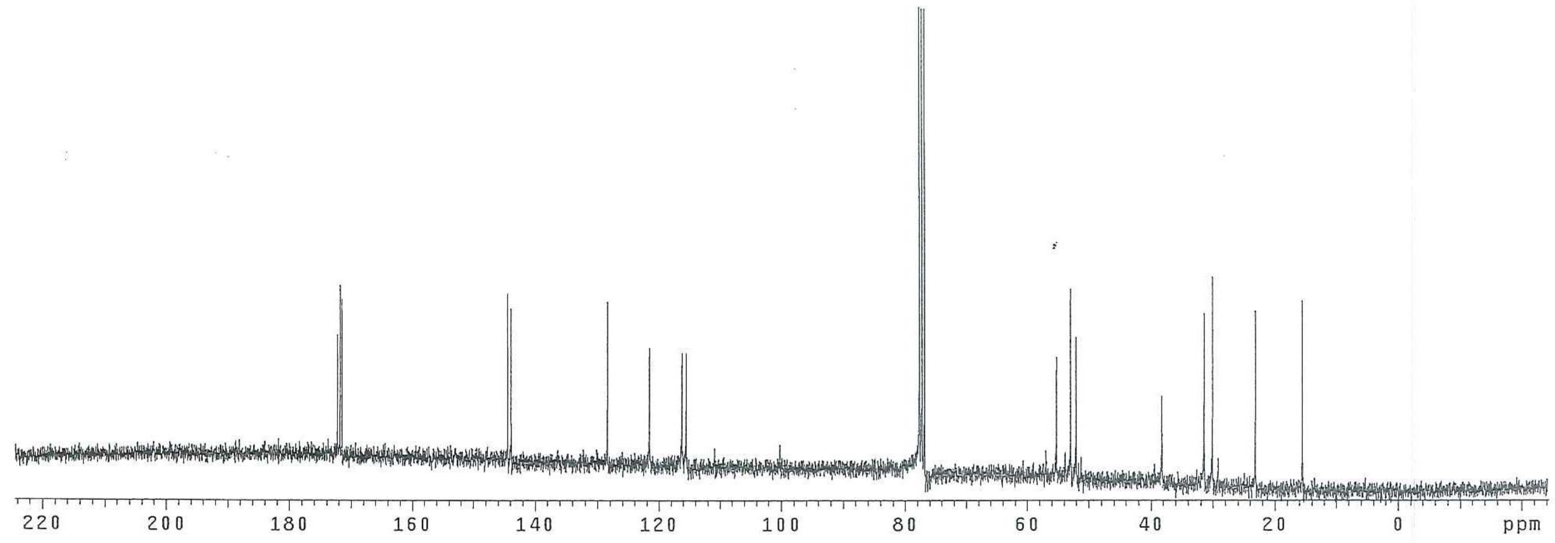

SP5 #475 RT: 10.47 AV: 1 NL: 1.71E5
T: + c ESI Full ms [50.00-1000.00]

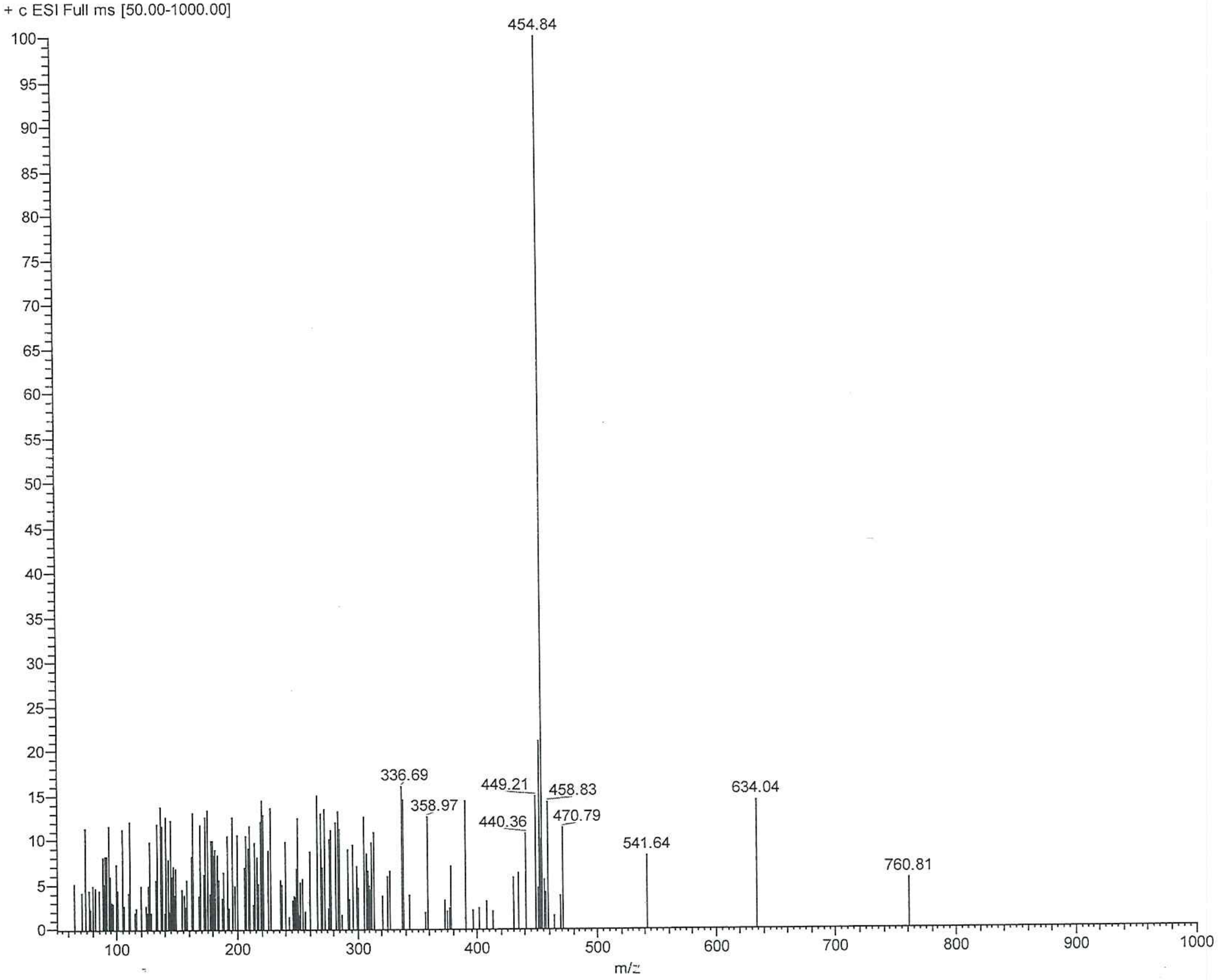

SP6

STANDAPJ 1H OBSERVE

exp1 std1h

SAMPLE		DEC. & VT	
date	May 16 2007	dfrq	300.059
solvent	CDCl3	dn	H1
file	exp	dpwr	41
ACQUISITION		dof	0
sfrq	300.059	dm	nnn
tn	H1	dmm	c
at	1.998	dmf	11400
np	17984	PROCESSING	
sw	4500.5	wtfile	
fb	2600	proc	ft
bs	16	fn	not used
tpwr	57		
pw	7.0	werr	
d1	1.000	wexp	
tof	0	wbs	
nt	16	wnt	
ct	16		
alock	n		
gain	20		
FLAGS			
il	n		
in	n		
dp	y		
DISPLAY			
sp	-804.1		
wp	4500.5		
vs	200		
sc	0		
wc	250		
hzmm	4.00		
is	500.00		
rfl	804.1		
rfp	0		
th	20		
ins	100.000		
nm cdc ph			

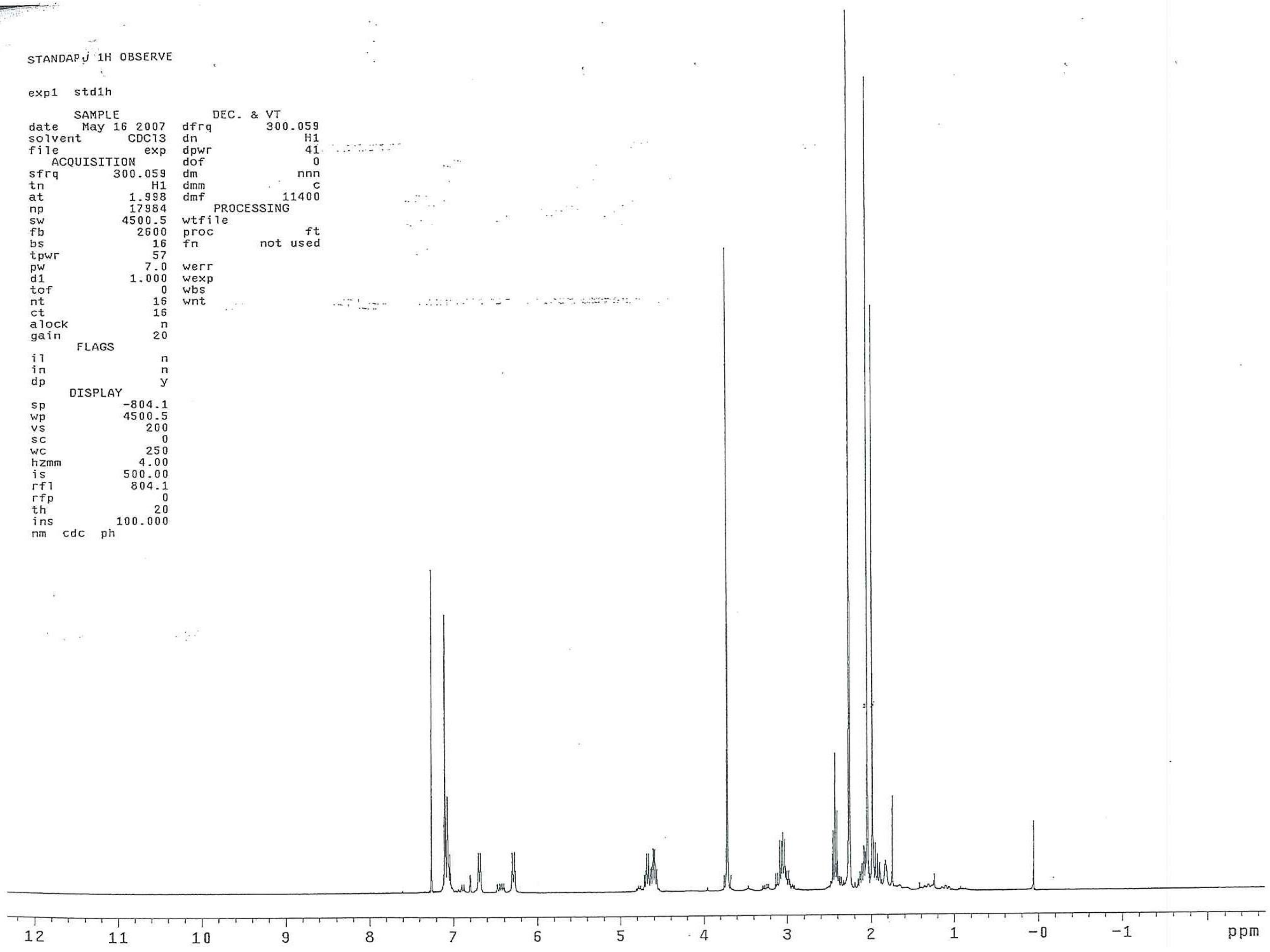

SP6

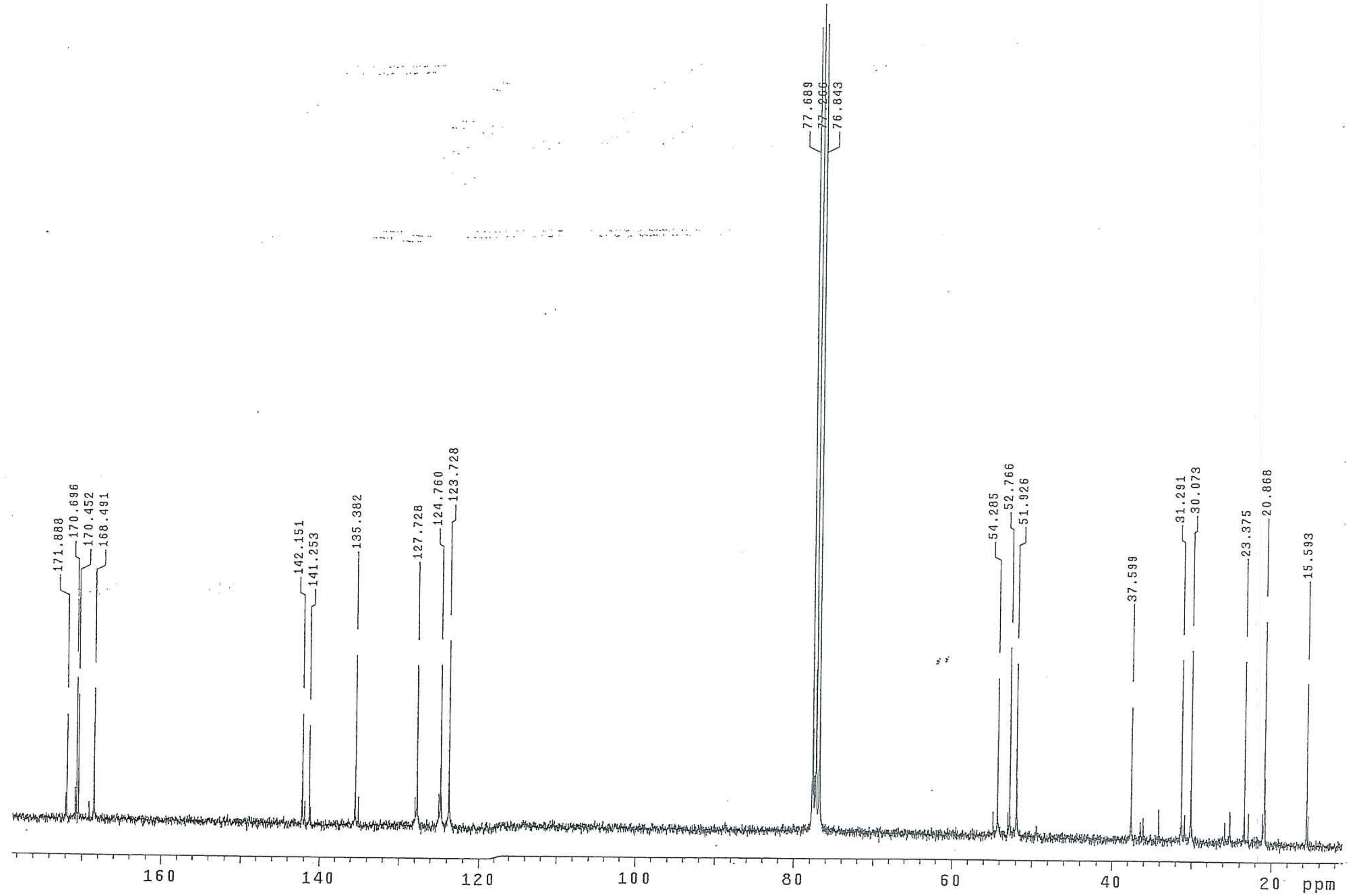

171.888
170.696
170.452
168.491
142.151
141.253
135.382
127.728
124.760
123.728
77.689
77.266
76.843
54.285
52.766
51.926
37.599
31.291
30.073
23.375
20.868
15.593
160
140
120
100
80
60
40
20
ppm

SP6 #202 RT: 4.69 AV: 1 NL: 1.28E6
T: + c ESI Full ms [50.00-1000.00]

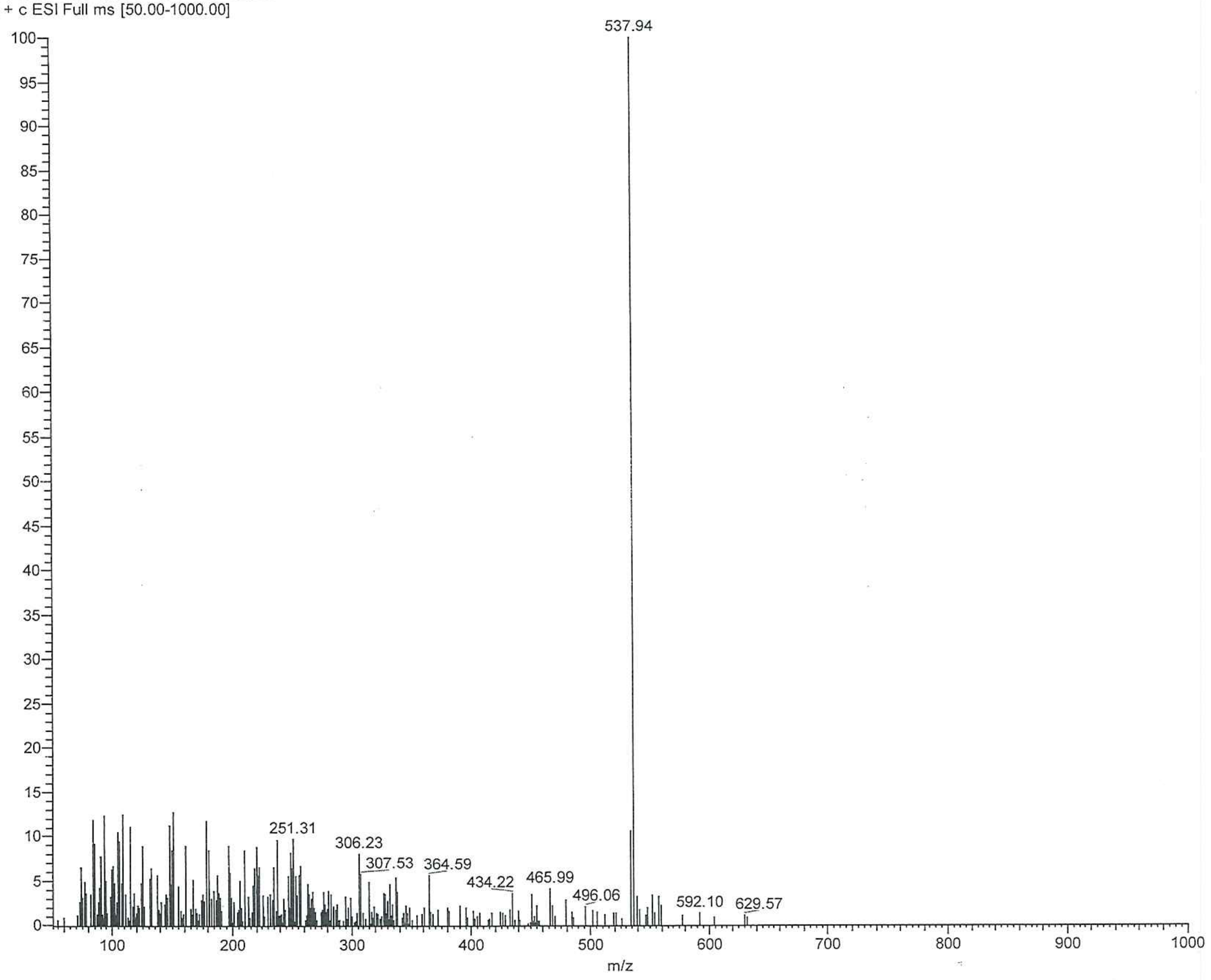

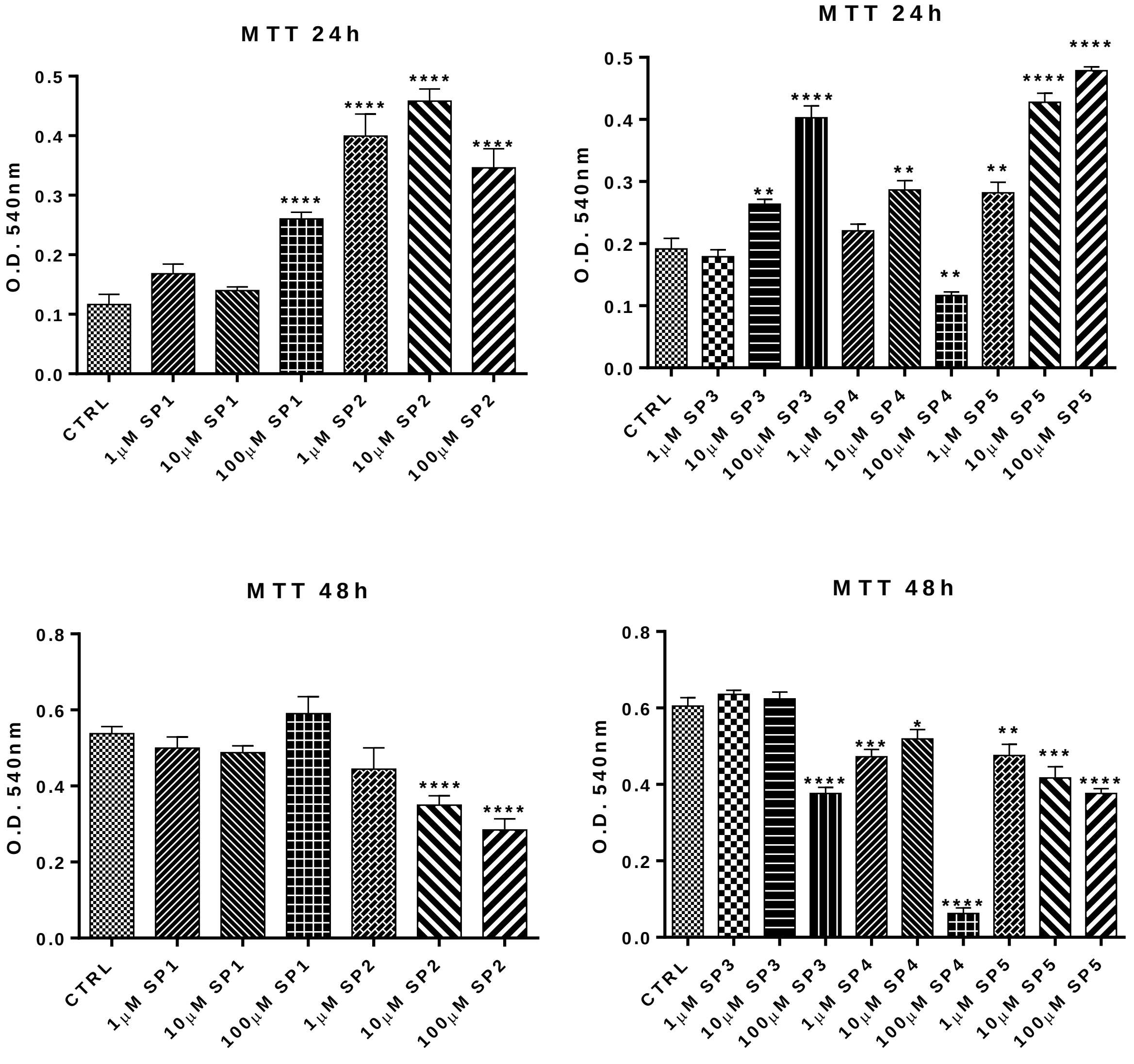

Figure 1S. Dose-response effects of LD and **SP1–5** in undifferentiated SH-SY5Y human neuroblastoma cells. MTT reduction assay in undifferentiated SH-SY5Y human neuroblastoma cells in the presence of LD and **SP1–5**. The cells were incubated for 24 or 48 h with increasing concentrations (1, 10, and 100 μM) of the compounds. After this period, cell viability was quantified by measuring the MTT reduction. CTRL: control without compounds. The means ± SEM derived from three different experiments (each with n = 16; **** $p < 0.0001$, *** $0.0001 < p < 0.0005$; ** $0.0005 < p < 0.001$; * $0.001 < p < 0.05$; n.s., $p > 0.05$).

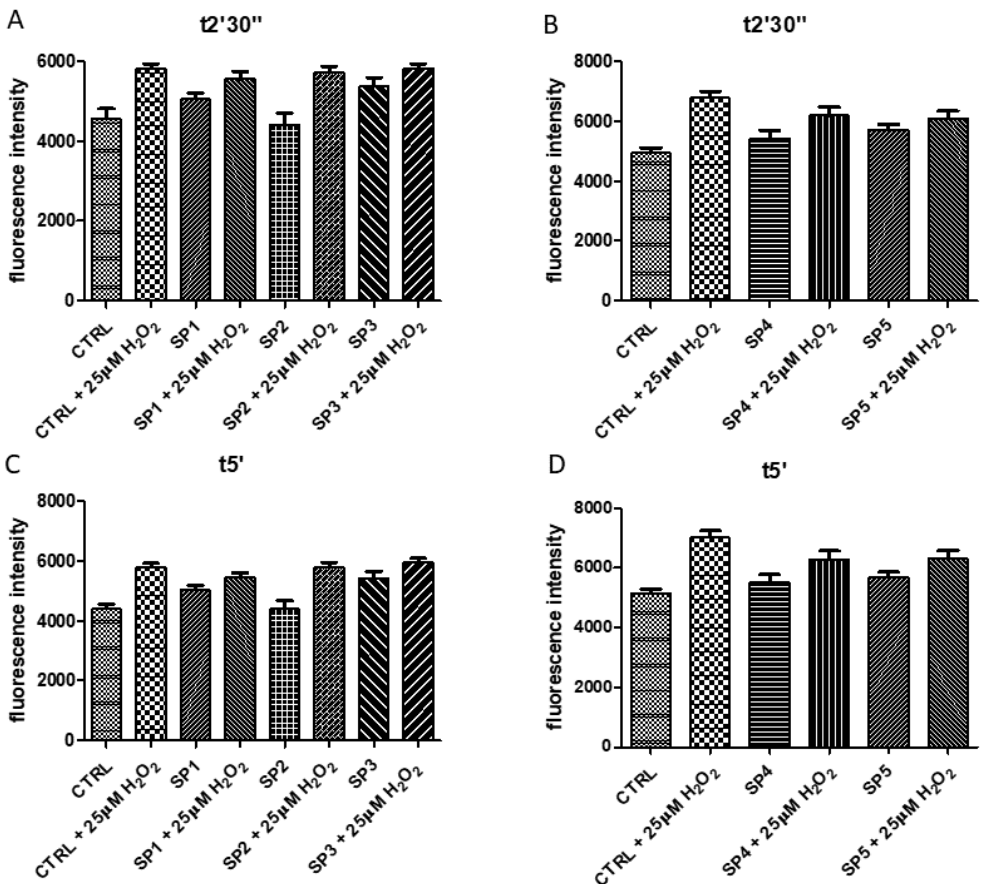

Figure 2S. Measurement of intracellular reactive oxygen species (ROS). The differentiated SY-SH5Y cells incubated with 1μM **SP1–3** (panel A and C) or **SP4–5** (panel B and D) for 24 h, were treated with 25 μM H_2O_2 for 5 min. The fluorescence intensities are reported in this figure at two points ($t_{2'30''}$ and $t_{5'}$) during the time-course. The means ± SEM were derived from two different experiments (each with n = 8; n.s., $p > 0.05$).
